# Mining and validation of novel genotyping-by-sequencing (GBS)-based simple sequence repeats (SSRs) and their application for the estimation of the genetic diversity and population structure of coconuts (*Cocos nucifera* L.) in Thailand

**DOI:** 10.1038/s41438-020-00374-1

**Published:** 2020-10-01

**Authors:** Kanamon Riangwong, Samart Wanchana, Wanchana Aesomnuk, Chatree Saensuk, Phakchana Nubankoh, Vinitchan Ruanjaichon, Tippaya Kraithong, Theerayut Toojinda, Apichart Vanavichit, Siwaret Arikit

**Affiliations:** 1 Department of Biotechnology, Faculty of Engineering and Industrial Technology, Silpakorn University, Sanamchandra Palace Campus, Nakhon Pathom, 73000 Thailand; 2 National Center for Genetic Engineering and Biotechnology (BIOTEC), National Science and Technology Development Agency (NSTDA), Khlong Luang Pathum Thani, 12120 Thailand; 3 Center for Agricultural Biotechnology, Kasetsart University, Kamphaeng Saen Campus, Nakhon Pathom, 73140 Thailand; 4 Rice Science Center, Kasetsart University, Kamphaeng Saen Campus, Nakhon Pathom, 73140 Thailand; 5 Chumphon Horticultural Research Center, Department of Agriculture, Bangkok, 10900 Thailand; 6 Department of Agronomy, Faculty of Agriculture at Kamphaeng Saen, Kasetsart University, Kamphaeng Saen Campus, Nakhon Pathom, 73140 Thailand

**Keywords:** Next-generation sequencing, Plant sciences

## Abstract

Coconut (*Cocos nucifera* L.) is an important economic crop in tropical countries. However, the lack of a complete reference genome and the limitations of usable DNA markers hinder genomic studies and the molecular breeding of coconut. Here, we present the results of simple sequence repeat (SSR) mining from a high-throughput genotyping-by-sequencing (GBS) study of a collection of 38 coconut accessions. A total of 22,748 SSRs with di-, tri-, tetra-, penta- and hexanucleotide repeats of five or more were identified, 2451 of which were defined as polymorphic loci based on locus clustering in 38 coconut accessions, and 315 loci were suitable for the development of SSR markers. One hundred loci were selected, and primer pairs for each SSR locus were designed and validated in 40 coconut accessions. The analysis of 74 polymorphic markers identified between 2 and 9 alleles per locus, with an average of 3.01 alleles. The assessment of the genetic diversity and genetic relationships among the 40 coconut varieties based on the analysis of population structure, principal coordinate analysis (PCoA), and phylogenetic tree analysis using the 74 polymorphic SSR markers revealed three main groups of coconuts in Thailand. The identified SSR loci and SSR markers developed in this study will be useful for the study of coconut diversity and molecular breeding. The SSR mining approach used in this study could be applied to other plant species with a complex genome regardless of the availability of reference genome.

## Introduction

Coconut (*Cocos nucifera* L.) is one of the most important economic crops in many tropical countries^1^. It is regarded as the “tree of life” and a symbol of the tropics and presents economic value because of its myriad edible and inedible products. Coconut is the only species of the genus *Cocos* in the family Aracaceae (Palmaceae). It is a dioecious plant (2n = 2x = 32) with an ~2.4 Gb haploid genome^2^. Coconut is native to coastal areas of Melanesia and southeast Asia, probably Malaysia, Indonesia (Moluccas Islands), Philippines, and Papua New Guinea^3^. It is widely distributed in tropical and subtropical regions of the world in over 80 countries across Asia, Africa, America, and Oceania^3^. Based on plant morphology and breeding habits, coconut is classified into two ecotypes: “tall” (*typica*) and “dwarf” (*nana*)^4^. Tall coconuts are commonly grown for commercial purposes and can be divided into two major groups: Pacific and Indo-Atlantic^5^. Dwarf coconuts are native to the Pacific region and cultivated worldwide, typically near human dwellings^1^. Tall coconut palms are predominately outcrossing and exhibit varying degrees of heterozygosity, while dwarf palms are normally self-pollinating and present higher levels of homozygosity, showing common morphological characteristics such as dwarf stature due to short internodes and slow growth of height, a slender trunk, a smaller crown, and large numbers of relatively small nuts with a low copra content^6^. Traditionally, genetic diversity assessment in coconut is based on morphological trait characterization and coconut breeding is performed through conventional methods, which are laborious, time consuming, and inefficient due to environmental factors and the limited number of phenotypic markers available^7,8^. DNA markers could help to overcome these limitations, as they are abundant and highly polymorphic and are not influenced by the environment.

The development of DNA markers in coconut presents potential for application to molecular breeding through marker-assisted selection (MAS). A number of DNA markers have been used to characterize genetic diversity in coconut, such as restriction fragment length polymorphisms (RFLPs) that defined two genetically distinct groups of tall coconut palms^9^, randomly amplified polymorphic DNA (RAPD) markers that revealed a moderate level of genetic diversity of 17 distinct South Pacific populations^10^, amplified fragment length polymorphisms (AFLPs) that revealed more variation in tall varieties (typica), rather than intermediate (*aurantiaca*) and dwarf (*nana*) varieties^11^, and simple sequence repeats (SSRs) that also defined two subgroups within tall coconuts^12,13^. SSRs or microsatellites are tandem DNA repeats of 1–6 nucleotides per unit located mostly in noncoding regions of eukaryotic genomes^14^. SSRs are useful for developing DNA markers because they are abundant, highly polymorphic, multiallelic, and codominantly inherited^15^. Noncoding SSRs are widely used to analyze genetic diversity and population structure^12,13,16,17^, construct linkage maps^18–20^, and detect quantitative trait loci^21–23^, while SSRs located in coding and untranslated regions may be effective functional markers^24^. Although SSR markers have been proven to be useful in coconut research for decades, the number of validated SSR markers is currently limited.

Despite the many advantages of SSR markers, genomic SSR identification and subsequent marker conversion were once expensive and time-consuming techniques^25^. Due to the advent of next-generation sequencing technologies, genomic SSR mining and marker development are currently fast and inexpensive. Because of its adaptability, high-throughput genotyping-by-sequencing (GBS) has had a significant impact on plant breeding and genetic research^26,27^. This approach can provide accurate results regardless of the target species, and does not require previous genomic information. In this study, we identified SSRs and developed new SSR markers using coconut genomic sequencing data from Illumina GBS. A total of 2451 SSR loci were identified, and PCR primers for these loci were obtained. Three hundred and fifteen of these markers were proposed as a potential set of SSR markers based on marker diversity. One hundred pairs of PCR primers were synthesized for the selected loci and tested in 40 diverse coconut accessions; 74 markers were shown to be polymorphic. These markers were proven to be useful for evaluating the genetic diversity of coconut accessions in Thailand and could be applied to other coconut germplasms.

## Results

### GBS sequencing

**Fig. 1:**
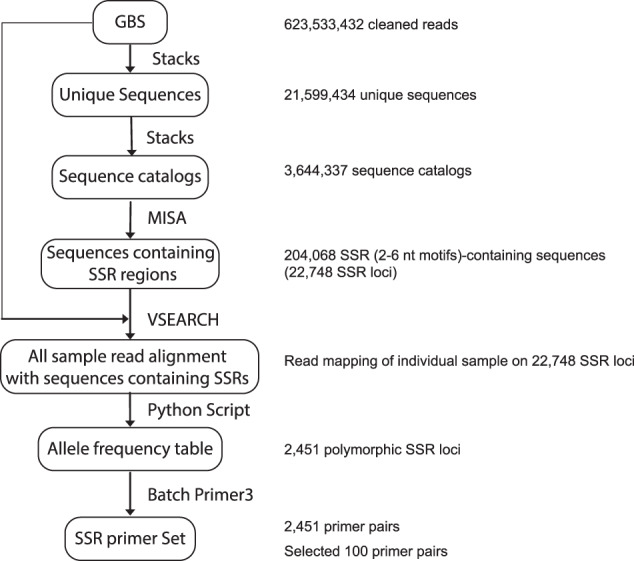
Bioinformatic workflow for GBS-based SSR sequence identification. The software packages used in each step are provided nearby the arrows. The types of input/output are provided in rounded rectangles and the numbers are listed on the right.

For the 38 coconut accessions, *Ape*KI-GBS libraries were constructed, and 100-bp long GBS reads were subsequently generated. In these samples, the total GBS raw reads ranged from 8.71 million to 28.14 million, with total nucleotides ranging from 0.84 Gb to 2.72 Gb. GBS sequence analysis and microsatellite mining were carried out using a modified GBS analysis workflow without using a reference genome (Fig. 1). After filtering low-quality reads and reads that lacked enzyme cutting sites by the *process_radtags* component of Stacks v1.39 software^28^, the total number of clean reads retained in the 38 libraries ranged from 8.62 to 27.89 million, with an average of 16.41 million. The total clean-read nucleotides ranged from 0.83 Gb to 2.69 Gb, with an average of 1.59 Gb, equivalent to 0.66x coconut genome coverage (2.42 Gb) (Table 1). The total number of filtered reads of all 38 samples was ~624 million reads, and the number of unique reads after reduction by *ustacks* was ~21.6 million reads. All the unique sequences were incorporated into sequence catalogs using *cstacks*, resulting in 3,644,337 consensus locus sets.

**Table 1:** Summary of sequencing reads generated for each of 38 coconut accessions.

Accession Code	Name	Raw reads (million)	Raw nucleotides (Gb)	Clean reads (million)	Clean nucleotides (Gb)	Genome Coverage	Unique reads	Reads containing SSRs
ACC.01	Ma Phraeo #1	12.93	1.26	12.81	1.25	0.52	707,077	4597
ACC.02	Nam Wan #1	16.31	1.59	16.14	1.57	0.65	507,313	5319
ACC.03	Thung Kled	13.78	1.35	13.65	1.33	0.55	464,790	4685
ACC.04	Pak Chok #1	14.44	1.41	14.3	1.39	0.58	495,496	5053
ACC.05	Papua New Guinea Brown Dwarf	18.82	1.84	18.63	1.82	0.75	537,061	5772
ACC.06	Cameroon Yellow Dwarf	15.33	1.5	15.18	1.48	0.61	453,127	4950
ACC.07	Rennell Island Tall	13.51	1.32	13.37	1.3	0.54	475,428	4772
ACC.08	West African Tall	10.65	1.04	10.55	1.03	0.42	434,731	4748
ACC.09	Kalok	14.11	1.38	13.97	1.36	0.56	505,999	5338
ACC.10	Thalai Roi	14.01	1.37	13.88	1.35	0.56	466,724	5185
ACC.11	Tahiti Tall	13	1.27	12.87	1.25	0.52	457,534	5176
ACC.12	Pak Chok #2	22.73	2.21	22.5	2.18	0.9	627,073	7098
ACC.13	Mu Si Som	20.27	1.97	20.07	1.95	0.8	541,446	5399
ACC.14	Nam Hom #1	17.17	1.67	17	1.65	0.68	541,769	5491
ACC.15	Nok Khum	17.07	1.66	16.91	1.64	0.68	547,604	5800
ACC.16	Nali-ke	11.31	1.1	11.19	1.09	0.45	417,957	4268
ACC.17	Thailand Tall Nakhon Si Thammarat	22.59	2.2	22.37	2.17	0.9	581,591	6754
ACC.18	Thailand Tall Thap Sakae	14.37	1.4	14.23	1.38	0.57	458,927	5032
ACC.19	Sri Lanka Tall	25.93	2.52	25.68	2.49	1.03	591,177	6679
ACC.20	Thailand Tall Sawi #1	24.64	2.39	24.4	2.37	0.98	644,324	7770
ACC.21	Thailand Tall Sawi #2	18.55	1.8	18.38	1.78	0.74	561,531	6075
ACC.22	Thailand Tall Sawi #3	22.51	2.19	22.29	2.16	0.89	583,280	6725
ACC.23	Mu Si Luang	14.1	1.36	13.97	1.35	0.56	598,930	4540
ACC.24	MaWa	8.71	0.84	8.62	0.83	0.34	379,036	4156
ACC.25	King coconut	9.28	0.9	9.19	0.89	0.37	405,583	3753
ACC.26	Thailand Tall Ko Samui	13.68	1.32	13.55	1.31	0.54	488,284	5007
ACC.27	Thailand Tall Ko Pha-ngan	16.35	1.58	16.19	1.56	0.65	505,773	5425
ACC.28	NDK	21.77	2.1	21.3	2.05	0.85	681,038	6249
ACC.29	YDK	14.11	1.36	13.97	1.35	0.56	504,694	4955
ACC.31	Mu Si Nu	24.87	2.4	24.63	2.38	0.98	988,313	6405
ACC.32	Maphrao So #1	20.24	1.96	20.05	1.93	0.8	546,293	6034
ACC.33	Ratchaburi 2	28.14	2.72	27.89	2.69	1.11	1,276,794	6925
ACC.34	Ratchaburi 3	13.52	1.3	13.41	1.29	0.53	695,301	4588
ACC.35	Ratchaburi 1	16.24	1.56	16.09	1.54	0.64	713,907	4955
ACC.37	Thailand Tall Ko Chang	8.91	0.86	8.83	0.85	0.35	465,241	3708
ACC.38	Maphrao Teun Dok	16.34	1.57	16.19	1.55	0.64	721,304	4777
ACC.39	Maphraeo #2	13.39	1.29	13.2	1.27	0.52	471,958	4812
ACC.40	Nam Hom #2	16.24	1.56	16.09	1.54	0.64	555,026	5093
	avg	16.58	1.61	16.41	1.59	0.66	568,406	5370
	Max	28.14	2.72	27.89	2.69	1.11	1,276,794	7770
	Min	8.71	0.84	8.62	0.83	0.34	379,036	3708
	Total	629.92	61.12	623.54	60.37	24.96	21,599,434	204,068

### Coconut SSR locus identification and the frequency and distribution of SSRs

**Fig. 2:**
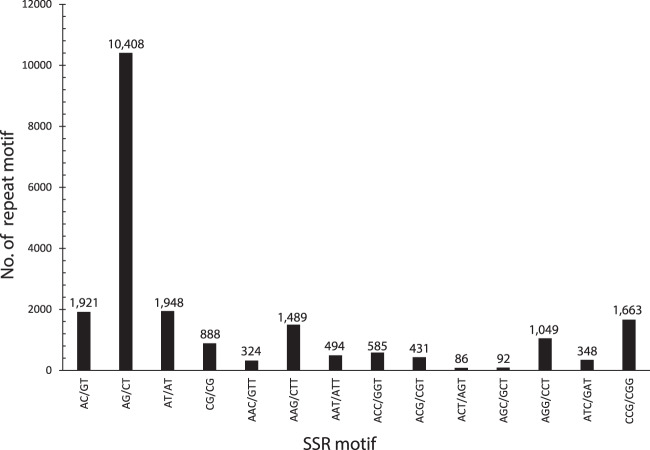
Frequency distribution of dinucleotide and trinucleotide SSRs. Numbers of SSR loci are listed based on their motifs.

We used the MIcroSAtellite identification tool (MISA)^29^ with the default setting to identify SSR-containing regions among the 21,599,434 unique sequences. The search for perfect SSR-containing regions was restricted to motifs of di-, tri-, teta-, penta-, and hexanucleotides. As a result, a total of 204,068 sequences containing 22,748 loci of SSR motifs were identified (Fig. 1). This collection of SSR loci consisted of 15,165 dinucleotide repeats (66.67%), 6570 trinucleotide repeats (28.88%), 659 tetranucleotide repeats (2.90%), 116 pentanucleotide repeats (0.51%) and 238 hexanucleotide repeats (1.05%) (Table 2). Dinucleotide repeats were identified as the most abundant microsatellite class (15,165 regions), followed by trinucleotide (6570) and tetranucleotide repeats (659). The assessment of the nucleotide composition of the repeat motifs of the two most abundant classes (dinucleotide repeats and trinucleotide repeats) revealed that the most frequent type of dinucleotide repeat was AG/CT (10,408 motifs), representing 68.63% of the total dinucleotides, while the most common type of trinucleotide repeat was CCG/CGG (1663 motifs), representing 25.31% of the trinucleotides (Fig. 2).

**Table 2:** Summary of coconut SSRs identified based on GBS sequences.

SSR motifs	Total number of identified SSR repeats								Frequency (%)
	5	6	7	8	9	10	>10	Total	
Dinucleotide		3678	2101	1708	1394	1126	5158	15,165	66.67
Trinucleotide	3596	1392	701	370	233	103	175	6,570	28.88
Tetranucleotide	399	141	57	36	19	2	5	659	2.90
Pentanucleotide	79	17	8	3	6	1	2	116	0.51
Hexanucleotide	142	47	13	16	7	6	7	238	1.05
Total	4221	5281	2887	2141	1668	1248	5347	22,748	100.00

### Coconut SSR primer design and marker validation

For each individual coconut accession, we used VSEARCH^30^ to build SSR locus clusters and used a custom Python script to generate an allele frequency table and identify polymorphic SSR loci. As a result, a total of 2451 putative polymorphic SSR loci were identified, and flanking primers for each locus were subsequently designed by using BatchPrimer3 (version 2.3.7)^31^ (Supplementary Information; Table S1). Among these sequences, 315 were selected as suitable for SSR primer design based on the following criteria: (1) the expected PCR product size was 80–100 bp, and (2) the selected loci were supported by GBS sequences from at least 20 coconut accessions. (Supplementary Information; Table S1). We randomly selected 100 of those 315 primer pairs to be validated by PCR. These 100 SSR loci included 74 dinucleotide repeats, 21 trinucleotide repeats, two tetranucleotide repeats, two pentanucleotide repeats, and one hexanucleotide repeat (Supplementary Information; Table S2).

**Fig. 3:**
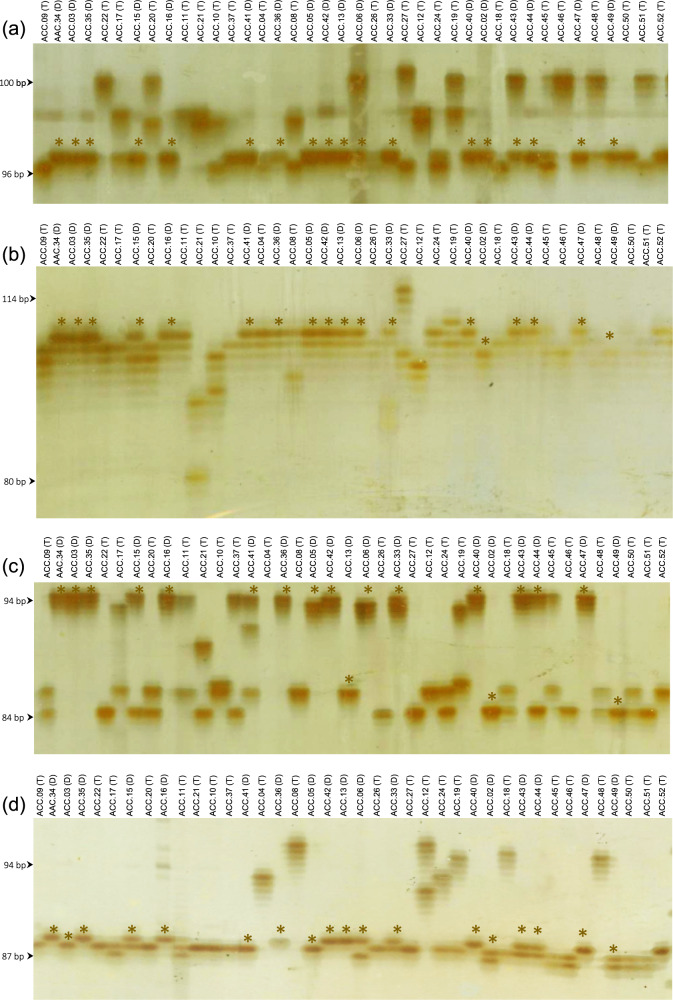
An illustration of PAGE gels showing allelic variation for high polymorphic SSR markers. **a** CnSSR5, **b** CnSSR9, **c** CnSSR16, **d** CnSSR28. The tall and dwarf types are denoted as (T) and (D), respectively. The asterisk (*) indicates the DNA bands of dwarf accessions.

To validate the efficacy of the newly developed SSR markers, 40 diverse coconut accessions, including both tall and dwarf coconut palms, were used in the analysis with the 100 SSR markers (Supplementary Information; Table S3). Based on the polyacrylamide gel electrophoresis (PAGE) results, we found that 74 out of 100 SSR markers clearly showed polymorphic patterns, presenting consistent, interpretable amplified products (Supplementary Information; Fig. S1). Among these markers, the SSR markers CnSSR5, CnSSR9, CnSSR16, and CnSSR28 were the five most polymorphic, exhibiting 5, 9, 6, and 7 alleles, respectively, in the 40 coconut genotypes (Fig. 3). An allele of the markers CnSSR16 (94 bp) was probably shared among Thai dwarf coconut accessions. Considering tall and dwarf accessions separately, all 74 SSR markers were polymorphic among all tall coconut accessions, but only 52 of them were polymorphic among the 18 dwarf coconut accessions (Table 3).

**Table 3:** List of primer pairs and genetic diversity information.

Locus Name	Primer Sequences (5′–3′)	Repeat Motif	Tm	Product size (bp)	No. of alleles			MAF	He			Ho			PIC		
					All	Tall	Dwarf		All	Tall	Dwarf	All	Tall	Dwarf	All	Tall	Dwarf
CnSSR1	F - GCATGCACGAATAAGGATA R - CTGAACTGAGATTCAAGAGGA	(TA)6	55	90	2	2	2	0.86	0.24	0.33	0.10	0.23	0.32	0.11	0.21	0.27	0.10
CnSSR2	F - CCAACCAAGCCACCATTA R - TGACCTCGATTGTGAATTTTA	(CT)9	56	82	3	3	1	0.91	0.17	0.31	0.00	0.08	0.16	0.00	0.16	0.27	0.00
CnSSR3	F - TCTGGTCCTCAAAAGGTGT R - GAAGCTGGAACAGTAGAAACA	(TC)12	55	96	4	4	2	0.77	0.37	0.50	0.10	0.26	0.38	0.11	0.32	0.42	0.10
CnSSR4	F - CAGCAGGTGCAAATAGTTTT R - TTTGACAAAGTCTCACCAGAT	(AG)11	55	83	3	3	2	0.85	0.27	0.42	0.05	0.13	0.19	0.06	0.25	0.38	0.05
CnSSR5	F - AGAGGGAAAGAGAGATTATGG R - AAGCCTTGTAAACCAAAGG	(GA)10	54	88	5	5	3	0.54	0.65	0.78	0.16	0.35	0.50	0.17	0.61	0.74	0.15
CnSSR8	F - CCAATGTATTGTGAGATGGAG R - TTCTTCTTCTCTTTTCGCTCT	(AG)10	55	82	3	2	3	0.86	0.24	0.33	0.11	0.23	0.32	0.11	0.22	0.27	0.10
CnSSR9	F - CAGCAGAGTAGACCTATTTTAT R - GTGTGTGTTTCTTGGTCTACA	(AG)21	55	100	9	9	3	0.49	0.64	0.65	0.29	0.05	0.09	0.00	0.57	0.62	0.27
CnSSR10	F - GCTTTAGGTCTCCTCACTTCT R - ATGTAAATAAAACCCCCTGTG	(TC)8	55	80	2	2	1	0.81	0.3	0.45	0.00	0.08	0.14	0.00	0.26	0.35	0.00
CnSSR12	F - AGCAACATTTGAGGTTTATATG R - AACTCTCTTCCACCTTGTAGG	(GA)6	54	91	2	2	2	0.65	0.46	0.46	0.44	0.3	0.27	0.33	0.35	0.36	0.35
CnSSR13	F - TGGGCTCTGTTTCCGAAC R - CACCCTCCAATCCCTCTC	(GA)10	58	80	3	3	2	0.84	0.28	0.40	0.10	0.13	0.23	0.00	0.26	0.37	0.10
CnSSR14	F - AGCCTAGTCAAGGAAATAAGC R - AGGATTTATCTCTTTTGCATGT	(AT)6	54	82	2	2	1	0.94	0.12	0.20	0.00	0.08	0.14	0.00	0.11	0.18	0.00
CnSSR16	F - CTTTGTTTTTCCCCTATTTGT R - ACTCTGGTGTAGGTGCAAAG	(TC)11	55	84	6	6	4	0.33	0.73	0.66	0.54	0.41	0.62	0.17	0.68	0.60	0.51
CnSSR17	F - AGCCATGCACCGTGGAAT R - AAGAAGAACTCCCAAACCAC	(CT)10	58	91	2	2	2	0.79	0.33	0.28	0.38	0.1	0.14	0.06	0.27	0.24	0.30
CnSSR18	F - AGCACATTCTCAGAAGAAAAA R - GCACAAGGATATGAATAACA	(TG)9	55	99	2	2	1	0.95	0.1	0.17	0.00	0.1	0.18	0.00	0.09	0.15	0.00
CnSSR19	F - AGGGGCGTGGCTGTAGGT R - ACGAACCCGCACCCTACC	(GGT)5	60	90	2	2	1	0.95	0.1	0.17	0.00	0	0.00	0.00	0.09	0.15	0.00
CnSSR20	F - AAACTGGTGGGAGGTGTG R - AAATTAAAGGAAGTCTCAGCAC	(TC)10	55	90	2	2	2	0.6	0.48	0.35	0.48	0.2	0.18	0.22	0.36	0.29	0.36
CnSSR21	F - GCCTTAATGATCTCAACCTTAC R - CCTAACCTGCACTCTTGGA	(AT)6	55	92	3	3	2	0.79	0.34	0.39	0.28	0.23	0.32	0.11	0.29	0.33	0.24
CnSSR22	F - TCTTGTACCTATGCCACCTTA R - TATAAAACAGGAGCGGGTCTA	(CT)11	55	91	4	4	1	0.83	0.3	0.48	0.00	0.18	0.32	0.00	0.28	0.44	0.00
CnSSR23	F - TGATGTTTAAGGTTTGGTGTT R - ACCTATTTCGTTCTTACCTATT	(CT)11	55	92	3	3	3	0.78	0.37	0.46	0.20	0.38	0.59	0.11	0.33	0.39	0.19
CnSSR25	F - CTTTACTTAGCTGTGGAGCAA R - GATTGCTGTTTAGGTTTCG	(AT)8	54	96	4	4	2	0.71	0.46	0.58	0.22	0.26	0.36	0.13	0.43	0.53	0.19
CnSSR26	F - CCTGCAACAGAAGCAATC R - GATGGGATTCGTTTGAAAT	(AAT)9	55	90	2	2	1	0.96	0.07	0.13	0.00	0.08	0.14	0.00	0.07	0.12	0.00
CnSSR28	F - GGAGCTCTCACAAGTCAAATA R - GGTCCCATTTCTTCTTCTCTA	(AG)8	55	87	7	7	3	0.46	0.69	0.64	0.54	0.43	0.45	0.39	0.64	0.61	0.46
CnSSR29	F - CGTTCAACGAGGCAGGTT R - CTTTCCCTATTGGCAGTATTT	(GA)6	57	87	2	2	1	0.89	0.2	0.33	0.00	0.08	0.14	0.00	0.18	0.27	0.00
CnSSR31	F - CACCAGCAATTGAGACTCTAC R - CAACGATGATGAGGAAGC	(ACC)5	55	81	2	2	2	0.5	0.5	0.47	0.46	0.25	0.32	0.17	0.38	0.36	0.35
CnSSR32	F - AAGGGGCTTTGATGTAATAAT R - TATGGTAGGCTTTCTTTTTCC	(TA)10	55	87	3	3	1	0.9	0.19	0.32	0.00	0.05	0.10	0.00	0.18	0.29	0.00
CnSSR33	F - GAACCCACCAAAAAGAGAG R - TCCTTGCTGTACTACTTGCTC	(AG)11	54	95	5	5	3	0.58	0.6	0.72	0.20	0.35	0.50	0.17	0.55	0.67	0.19
CnSSR34	F - AAAACGCCAAAACCATTA R - TTGAAAGAAGCAGAAGAAGAA	(TTGT)3	54	83	3	3	3	0.55	0.59	0.61	0.32	0.33	0.55	0.06	0.52	0.53	0.29
CnSSR35	F - GGATCGGGCTGATCTTAT R - CGGATGAAGGCATGTATATTA	(TA)8	55	89	3	3	2	0.75	0.4	0.53	0.15	0.15	0.23	0.06	0.35	0.45	0.14
CnSSR36	F - CCCTAGCATTCAAACATACAT R - CGAGACAAATCGTACCCATA	(CT)12	55	97	4	4	3	0.65	0.52	0.65	0.20	0.3	0.45	0.11	0.46	0.58	0.19
CnSSR37	F - AGAGGGTTTGATGGAATAAAT R - AGGTATGGTCAGTCATTTTTG	(CAAAG)6	54	80	3	2	3	0.61	0.48	0.42	0.53	0.13	0.14	0.11	0.38	0.33	0.41
CnSSR38	F - CATGTACCTGCTCTCATTCAT R - CTATCAGAACCATCCAACATC	(TGT)5	55	88	2	2	2	0.66	0.45	0.45	0.44	0.18	0.23	0.11	0.35	0.35	0.35
CnSSR40	F - GCCAGCACAAGGGATATT R - GGAAAAGAGGATGAAGAAGAG	(TA)7	55	91	2	2	2	0.86	0.24	0.33	0.10	0.28	0.41	0.11	0.21	0.27	0.10
CnSSR42	F - CCAGAGTTTTCGTTTTGTTTT R - TTTGAACAGCCACACTCC	(CT)10	55	86	2	2	1	0.97	0.05	0.09	0.00	0.05	0.10	0.00	0.05	0.09	0.00
CnSSR44	F - CTAAGCGCTAAGATGATGAGA R - ATCGCCATCTCTCTCTCC	(GA)10	55	80	3	3	1	0.88	0.23	0.38	0.00	0.18	0.32	0.00	0.21	0.34	0.00
CnSSR46	F - TATCCAATCTCACCCCATT R - CTCTCTCATGAACGCAGAGT	(TC)16	55	86	3	3	3	0.66	0.5	0.62	0.25	0.2	0.23	0.17	0.45	0.55	0.23
CnSSR48	F - ATCACAATGCCTTTTGTACC R - TGGTTGAACTTAACTGTCTTCA	(TC)8	55	85	4	4	2	0.56	0.56	0.62	0.28	0.35	0.55	0.11	0.48	0.55	0.24
CnSSR49	F - CAGCCCTCTGATAGTCACC R - ACTGACATTGCAGAGAGAGAA	(AC)7	55	85	2	2	2	0.53	0.5	0.48	0.44	0.3	0.36	0.22	0.37	0.37	0.35
CnSSR50	F - AAATTACTGGATCCCCTACC R - AAGCCCTATCATCTTAACCTT	(AT)7	54	88	2	2	2	0.63	0.47	0.46	0.48	0.00	0.00	0.00	0.36	0.36	0.36
CnSSR51	F - TCAACCCTCAAAGTGATTCTA R - AAGGAAGAAAATCTGCATGAC	(TC)10	55	82	3	3	3	0.39	0.65	0.59	0.60	0.3	0.42	0.17	0.58	0.52	0.53
CnSSR52	F - ATGGTGCTCTCCCTCGAC R - GCTAACTCTTCCTTCGAAACT	(TTC)8	56	96	2	2	2	0.5	0.5	0.40	0.35	0.15	0.18	0.11	0.38	0.32	0.29
CnSSR53	F - CCATTTCTCTTGTCAACCTAC R - ATCAAAAGACCTATGCACAAA	(AT)11	54	80	4	4	2	0.81	0.32	0.49	0.05	0.23	0.36	0.06	0.3	0.45	0.05
CnSSR54	F - AACCATGGGCTCTCGACT R - ATGACGCAAGGAAAGCTC	(GA)13	55	81	3	3	2	0.59	0.54	0.62	0.35	0.18	0.14	0.22	0.46	0.54	0.29
CnSSR56	F - ATCGCACTCTTCCTCTCC R - GAGAAAACATGGGGCAAG	(CT)8	55	87	4	4	2	0.46	0.64	0.61	0.46	0.03	0.05	0.00	0.57	0.55	0.35
CnSSR57	F - CCGTCGTCAGTACCAAATTAT R - GTAGTCCCCAAGGAAGAGAG	(GA)10	55	80	2	2	2	0.68	0.44	0.49	0.05	0.25	0.41	0.06	0.34	0.37	0.05
CnSSR58	F - CCTGGAATCAACCATAATCTA R - TAAGCATGTTAATGCTCTCCT	(GTG)5	54	91	3	3	3	0.74	0.42	0.51	0.29	0.34	0.50	0.17	0.37	0.45	0.26
CnSSR62	F - CTGGGATCCTCAGTTGTTAAT R - AAGAAGATGACAAAGATTAGGT	(GTTTG)3	54	80	3	3	2	0.9	0.18	0.27	0.05	0.15	0.23	0.06	0.17	0.25	0.05
CnSSR63	F - GCAGCAGGAAGCAAATAATA R - CCTTCTTGAGCTTAGAGAAAAA	(AG)7	55	84	3	3	2	0.49	0.58	0.63	0.46	0.23	0.36	0.06	0.49	0.55	0.35
CnSSR64	F - TCGTAATAAAAAGGAGTACCG R - TTTCTTACTAGATGGGTCACG	(AG)9	54	82	2	2	2	0.6	0.48	0.44	0.50	0.34	0.41	0.28	0.36	0.34	0.37
CnSSR65	F - TGCAGAGATAGGAAGAGATAGAG R - CAACCAGAGGAGAGCAGAG	(TCC)6	55	82	2	2	2	0.93	0.14	0.20	0.05	0.15	0.23	0.06	0.13	0.18	0.05
CnSSR67	F - CGACTTCCCTAGTTCTTTTTC R - CTTTCTTTGTTTATGCTGGAA	(AT)9	55	82	3	3	2	0.77	0.36	0.46	0.16	0.26	0.32	0.18	0.3	0.38	0.15
CnSSR68	F - AGCACTTGAGATCAAAATGAA R - TACGTACACCACCTTTGATTC	(GGA)5	55	87	2	2	2	0.89	0.2	0.27	0.10	0.03	0.05	0.00	0.18	0.23	0.10
CnSSR69	F - ATTGCCGAGGCCGGTGGA R - AAGGTGAGGGAGAAGAAGAG	(TC)7	60	93	2	2	2	0.91	0.16	0.20	0.10	0.13	0.23	0.00	0.15	0.18	0.10
CnSSR71	F - CCTTTAGAGGTCGTCTCTCC R - GACACAATAGAGAGGGCAGA	(GAA)8	55	91	2	2	1	0.95	0.1	0.17	0.00	0.1	0.18	0.00	0.09	0.15	0.00
CnSSR72	F - TGAGTTTAACAGGGTGGTTAC R - GAGACAAGGCAGTCATCATAG	(AG)7	55	92	3	3	1	0.86	0.24	0.39	0.00	0.25	0.45	0.00	0.22	0.34	0.00
CnSSR73	F - CAGCTGGAGACAAGAATTAAG R - GGATCCTCAGTTGTTAATGG	(TTA)7	55	95	3	3	1	0.9	0.18	0.30	0.00	0.15	0.27	0.00	0.17	0.27	0.00
CnSSR77	F - ATTTTAGCTTTCTTGGATTCG R - GCAAGCATCAGATGTTATAG	(TC)9	54	83	2	2	1	0.78	0.35	0.48	0.00	0.1	0.18	0.00	0.29	0.37	0.00
CnSSR78	F - AGCCCTCCAACATCCTTG R - GAGAAAGAAGCAAAGAGAGAAA	(TC)8	57	97	5	5	1	0.87	0.24	0.40	0.00	0.13	0.24	0.00	0.23	0.39	0.00
CnSSR80	F - TGCTGTTGTTACTATTTCGATG R - TCATCCTCGAGGTCCTTAC	(GT)8	55	87	2	2	2	0.86	0.24	0.33	0.10	0.08	0.05	0.11	0.21	0.27	0.10
CnSSR82	F - CAGCGCCATAGGTTTATATG R - GAGCGGGATTTATGCAAT	(TAA)6	55	99	2	2	2	0.97	0.05	0.04	0.06	0.05	0.05	0.06	0.05	0.04	0.06
CnSSR84	F - TTATTATGATAGCGTGCACAT R - ATTTTCAAACATGGGTACAT	(CT)19	53	85	2	2	2	0.65	0.45	0.45	0.46	0.13	0.05	0.24	0.35	0.35	0.35
CnSSR85	F - CTTTGGACAAAATGCATGA R - AATTATCCACACACACACACA	(TC)7	55	84	5	4	3	0.78	0.36	0.41	0.29	0.28	0.33	0.22	0.32	0.36	0.27
CnSSR86	F - CTTCTTGTCCCTCTTTCACTC R - TAAAGGAATGCACCATCAAT	(AAAG)9	55	83	2	2	1	0.9	0.18	0.30	0.00	0.00	0.00	0.00	0.16	0.25	0.00
CnSSR87	F - TAGGTGCACAAGAATGTGAAT R - TTCTCATGTATTGTTTTCCTTCT	(AAC)4	55	93	5	5	1	0.79	0.36	0.55	0.00	0.25	0.45	0.00	0.33	0.50	0.00
CnSSR89	F - CATCAGCCACCTGAAAAA R - CATTAAATAGTCGGCTCCATC	(AG)10	55	93	2	2	1	0.95	0.1	0.17	0.00	0.05	0.09	0.00	0.09	0.15	0.00
CnSSR90	F - GCACTTGGTACCTTCAAATAA R - ATCACATAAATGCCAATTCAC	(CTT)6	55	85	4	2	4	0.94	0.12	0.04	0.21	0.1	0.05	0.17	0.12	0.04	0.20
CnSSR92	F - GCAGAGAAAGCACCATCTAAT R - TTGTCCTGTACGTTCTCTCTT	(TA)8	55	98	3	3	3	0.64	0.53	0.52	0.53	0.23	0.27	0.17	0.47	0.47	0.47
CnSSR93	F - AAGAGGATGGTAGGCATAAAC R - GCATACACTTGCTGTTGTCTA	(GA)6	55	81	4	4	4	0.44	0.69	0.67	0.41	0.33	0.36	0.28	0.63	0.62	0.37
CnSSR94	F - AAGAGCTCTAGATCTGGCAAT R - CTCCTTATTGATGGCCTTT	(AG)7	55	90	2	2	1	0.93	0.14	0.24	0.00	0.00	0.00	0.00	0.13	0.21	0.00
CnSSR95	F - CAATTTGCCTCCCTTAAAT R - TGCACCAACATAATTTACCA	(GT)10	55	91	3	3	1	0.9	0.18	0.30	0.00	0.1	0.18	0.00	0.17	0.27	0.00
CnSSR96	F - CATGGCATATCCAATATGTTT R - AGGAGTAACATGCATTTCTGT	(CGC)7	54	80	3	3	2	0.9	0.18	0.28	0.05	0.15	0.23	0.06	0.17	0.26	0.05
CnSSR97	F - CAAAGCCACCATCCCTTC R - CTACCGCTAGGCGACGAGGAG	(GGCTCA)5	60	87	3	3	3	0.69	0.47	0.48	0.44	0.58	0.59	0.56	0.41	0.40	0.40
CnSSR98	F - GGGCCAACCAATATAGCTC R - GGCTTAGGCGTCAATTTT	(GGA)7	55	90	4	4	2	0.54	0.59	0.66	0.28	0.3	0.36	0.22	0.52	0.60	0.24
CnSSR99	F - ACGGAGGGGCAAATGGAC R - CCCGCCACCATCTCCTCT	(CT)7	60	83	2	2	2	0.81	0.31	0.39	0.20	0.18	0.24	0.11	0.26	0.31	0.18
CnSSR100	F - CATCATCCTCTCTTTTCCTTC R - GATTCGGCCTTTCAAATC	(CGG)4	55	96	2	2	1	0.86	0.24	0.38	0.00	0.08	0.14	0.00	0.21	0.30	0.00
				Average	3.01	2.95	1.99	0.75	0.35	0.42	0.20	0.19	0.26	0.10	0.31	0.36	0.17

*Ta* Annealing temperature, *Na* Allele number, *Ho* Observed Heterozygosity, *He* Expected heterozygosity, *PIC* polymorphism information content.

### Estimation of the genetic diversity and population structure of coconuts in Thailand using the newly developed SSR markers

To evaluate the utility of the newly developed SSR markers, we used these markers to characterize the genetic diversity of a collection of 40 coconut accessions consisting of 35 accessions of coconuts grown in Thailand collected from different locations across the country, and the other five accessions were foreign varieties present in the country (Supplementary Information; Table S3). The results of the genotyping analysis of the 40 coconut accessions with the 74 polymorphic SSR markers revealed 223 alleles. The number of observed alleles for all polymorphic SSR markers among all 40 coconut accessions ranged from 2 to 9, with an average of 3.01 alleles per locus; that among tall accessions ranged from 2 to 9, with an average of 2.95; and that among dwarf accessions ranged from 1 to 4, with an average of 1.99 alleles per locus. The estimated polymorphism information content (PIC) of the 74 markers for all 40 coconut accessions ranged from 0.05 to 0.68, with an average of 0.31; that for tall coconut accessions ranged from 0.04 to 0.74, with an average of 0.36; and that for dwarf coconut accessions ranged from 0 to 0.53, with an average of 0.17 (Table 3). The gene diversity (expected heterozygosity: He) for all coconut accessions ranged from 0.05 to 0.73, with an average of 0.35; that for tall coconut accessions ranged from 0.04 to 0.78, with an average of 0.42; and that for dwarf coconut accessions ranged from 0 to 0.60, with an average of 0.20. The observed heterozygosity (Ho) for all coconut accessions ranged from 0 to 0.58, with an average of 0.19; that for tall coconut accessions ranged from 0 to 0.62, with an average of 0.26; and that for dwarf coconut accessions ranged from 0 to 0.56, with an average of 0.10. We found that the CnSSR9 marker yielded the highest number of alleles (9 alleles), with a PIC of 0.57 (Table 3). Considering the heterozygous genotypes of each individual among the 40 accessions, the heterozygosity of dwarf accessions ranged from 1.35 to 31.08%, and that of tall accessions ranged from 15.05 to 47.95% (Supplementary Information; Table S4). The accessions exhibiting the lowest and highest heterozygosity among the dwarf accessions were ACC.34 (Ratchaburi 3) and ACC.44 (Khom), respectively. The accessions exhibiting the lowest and highest heterozygosity among the tall accessions were ACC.21 (Thailand tall Sawi #2) and ACC.24 (MaWa), respectively.

**Fig. 4:**
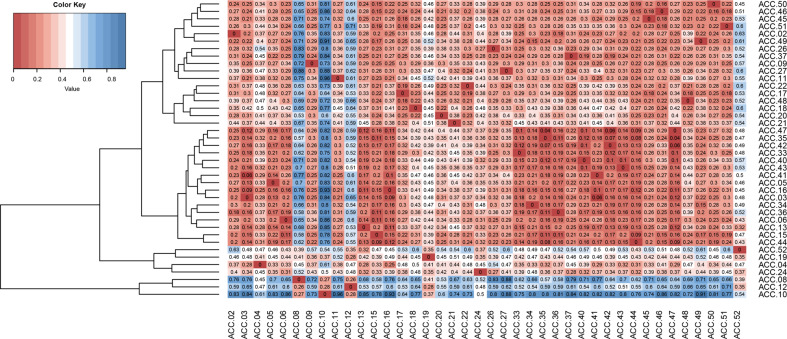
Heatmap hierarchical clustering of the genetic distance calculated based on dissimilarity matrix of 40 coconut accessions. A value of 1 corresponds to complete dissimilarity (blue) and a value of 0 indicates equivalent (red).

The genetic distance based on a dissimilarity matrix calculated from the 223 SSR alleles between each pair of the 40 coconut accessions ranged from 0.04 to 0.96, with an average of 0.35 (Fig. 4). The genetic distance within the group of dwarf coconut accessions was markedly lower than that within the group of tall coconut accessions, as the genetic distance of each pair among the dwarf accessions ranged from 0.04 to 0.30, with an average of 0.18, and that among the tall accessions ranged from 0.16 to 0.96, with an average of 0.42. The most distant accessions based on the dissimilarity matrix were ACC.08 (West African tall), ACC.10 (Thalai Roi) and ACC.12 (Pak Chok #2), followed by ACC.52 (Phuang Roi Si Thong), ACC.19 (Sri Lanka tall), ACC.4 (Pak Chok #1), and ACC.24 (MaWa: Malayan Yellow dwarf x West African tall).

**Fig. 5:**
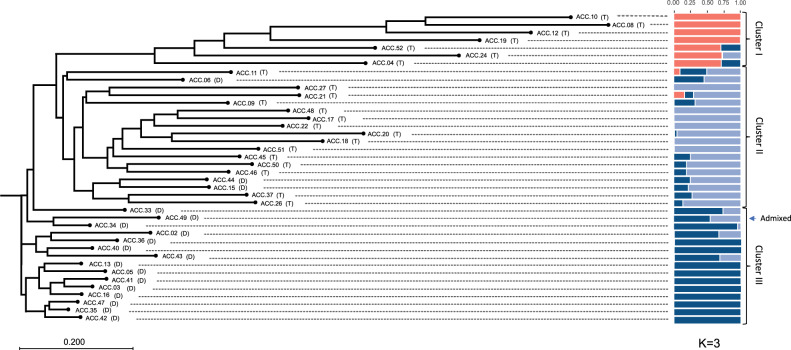
**Phylogenetic tree and population structure at*K*** = 3 for 40 coconut accessions. Each horizontal bar represents an individual and its assignment probability to belong to one cluster. Colors represent different assigned clusters. Tall and dwarf types are indicated by (T) and (D), respectively.

Based on the independent STRUCTURE analysis of the 40 accessions, the maximum delta *K* was detected at *K* = 3, indicating three subgroups (Fig. 5 and Supplementary Information; Fig. S2). For each *K*-value, genotypes with membership probability >60% were assigned to the same group, while those with < 60% probability in any group were assigned as “admixed”^32^. The three clusters of subpopulations among the 40 coconut accessions identified by STRUCTURE analysis were similar to those revealed by phylogenetic tree analysis (Fig. 5). Cluster I contained seven exclusively tall accessions, including the Thailand tall accessions ACC.04 (Pak Chok #1), ACC.10 (Thalai Roi), ACC.12 (Pak Chok #2) and ACC.52 (Phuang Roi Si Thong); two foreign tall accessions, ACC.08 (West African tall) and ACC.19 (Sri Lanka tall); and a hybrid accession, ACC.24 (MaWa). Among these, four accessions (ACC.08, ACC.10, ACC.12, and ACC.19) had pure genotypes, the other two (ACC.04 and ACC.52) exhibited mixed genotypes from Cluster I and Cluster III, and another accession (ACC.24) exhibited mixed genotypes from Cluster I and Cluster II.

Cluster II contained 18 coconut accessions, including 14 Thailand tall accessions, ACC.26 (Thailand tall Ko Samui), ACC.37 (Thailand tall Ko Chang), ACC.46 (Tha Nan), ACC.50 (Mu Si Mo), ACC.45 (Maprao So #2), ACC.51 (Maphrao Fai), ACC.18 (Thailand tall Thap Sakae), ACC.20 (Thailand tall Sawi #1), ACC.22 (Thailand tall Sawi #3), ACC.17 (Thailand tall Nakhon Si Thammarat), ACC.48 (Maphrao Fai Kathi), ACC.09 (Kalok), ACC.21 (Thailand tall Sawi #2), ACC.27 (Thailand tall Sawi #3), one foreign tall accession, ACC.11 (Tahiti tall), and three dwarf accessions, including two Thailand dwarf coconut accessions, ACC.44 (Khom) and ACC.15 (Nok Khum), and one foreign dwarf coconut, ACC.06 (Cameroon yellow dwarf). Among these, six accessions (ACC.17, ACC.18, ACC.22, ACC.27, ACC.48, and ACC.51) had pure genotypes. The rest of the accessions in this cluster mostly exhibited mixed genotypes from Cluster II and Cluster III, except for two accessions (ACC.11 and ACC.21), which exhibited mixed genotypes from all three clusters.

Cluster III was a homogenous genetic group of dwarf accessions containing 15 dwarf accessions, including 14 Thailand dwarf accessions, ACC.02 (Nam Wan #1), ACC.03 (Thung Kled), ACC.13 (Mu Si Som), ACC.16 (Nali-ke), ACC.35 (Ratchaburi 1), ACC.33 (Ratchaburi 2), ACC.34 (Ratchaburi 3), ACC.36 (Nam Wan #2), ACC.40 (Nam Hom #2), ACC.41 (Pathiu), ACC.42 (Nam Wan #3), ACC.43 (Nam Hom Kathi), ACC.47 (Nam Hom #3) and ACC.49 (Nam Wan #4), and one foreign dwarf coconut accession, ACC.05 (Papua New Guinea brown dwarf). Among these, ten accessions (ACC.03, ACC.05, ACC.13, ACC.16, ACC.35, ACC.36, ACC.40, ACC.41, ACC.42, and ACC.47) had pure genotypes. The rest of the accessions in this cluster (ACC.02, ACC.33, ACC.34, and ACC.43) exhibited mixed genotypes from Cluster II and Cluster III. The accession ACC.49 was considered admixed as the value of membership probability revealed by STRUCTURE was less than the threshold of 0.60.

**Fig. 6:**
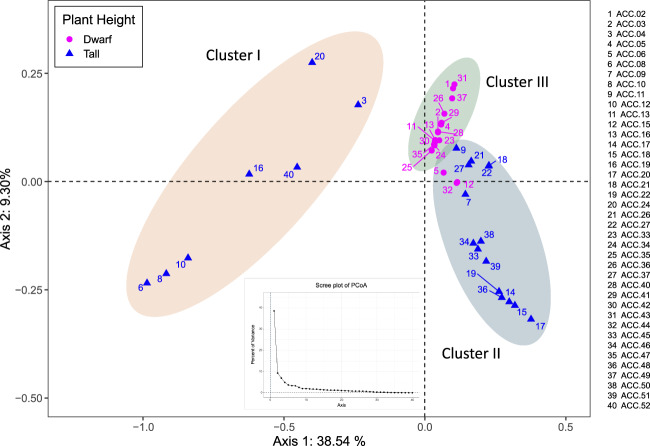
Principle coordinates analysis (PCoA) clustering 40 coconut accessions using Nei’s genetic distance based on 74 SSR markers. The x- and y-axes are indicated by the first and second coordinates, respectively, and the values show the percentages of variation explained. Circles and triangles indicate dwarf and tall accessions, respectively. Each cluster is indicated by an oval. Scree plot of PCoA is provided at the bottom of the figure.

The three clusters were also supported by principal coordinate analysis (PCoA) as analyzed by DARwin 6.0^33^software with 74 SSR markers. The total proportions of the variation explained by the first and second principal components were 38.54% and 9.30%, respectively (Fig. 6). Cluster I, exclusively containing tall coconuts, was clearly separated from the other two clusters. The number of SSR markers could be reduced to 49 to evaluate PCoA and achieve the same results (Supplementary Information; Fig. S3, Table S5). Discriminant Analysis of Principal Component (DAPC) was also performed using the *adegenet* package^34^ to cluster 40 coconut accessions. The results obtained from DAPC analysis supported the PCoA analysis (Supplementary Information; Fig. S4). We also performed a Mantel test using GenAlEx (6.51)^35^ to check the occurrence of a positive correlation (*r* > 0) between the Nie’s genetic distance and geographic distances among the 40 genotypes. As a result, the genetic and geographic distance were not significantly correlated (*r* = 0.12, *p* = 0.13; Supplementary Information; Fig. S5).

## Discussion

Coconut (*Cocos nucifera*) is a plant species with a large genome size (>2 Gb) in which no complete reference genome has been released. Therefore, studies of coconut genomics using a genome resequencing-based approach that requires a complete reference genome are limited. Microsatellites (SSRs) are currently markers of choice for several types of genetic diversity studies in coconut^36–40^. SSR loci have been conventionally identified through the laborious technique of genomic hybridization. The SSR markers developed based on this strategy include 296 validated SSR markers available at Tropgenedb (tropgenedb.cirad.fr). The SSR markers from this source have been frequently used in coconut genetic diversity studies^6,13,41^. Alternatively, with the advancement of next-generation sequencing, NGS-based approaches for identifying a large number of SSR loci have been proposed; for example, RNA sequencing (RNA-seq) analysis identified 6608 gene-based SSR loci from 57,304 unigenes^8^. From these loci, 309 primer pairs were developed, 191 of which were polymorphic across a set of ten coconut accessions. Recently, a total of 7139 genome-wide SSR markers were designed on the basis of a whole-genome assembly of coconut^42^. However, the validation of these markers was not reported. In this study, we applied a genotyping-by-sequencing (GBS) approach to generate DNA sequences from 38 different coconut accessions and subsequently identified coconut SSR loci from these GBS sequences. The restriction enzyme *Ape*KI, used in this study, has been widely used in the preparation of GBS libraries in several crops because it can produce an appropriate number of fragments for sequencing^43,44^. In addition, because of the methylation sensitivity of this enzyme, it can eliminate fragments generated from repetitive methylated genomic regions in the coconut genome, as coconut has been estimated to contain ~73% of repetitive sequences^2^. In fact, the *Ape*KI enzyme has been documented to show bias towards the coding areas. As a result, we identified 22,748 SSR loci, and flanking primers were designed for 2451 of them. A final set of 100 SSR loci were employed to develop SSR markers, and the markers were validated using 40 diverse coconut accessions collected from different parts of Thailand. Seventy-four of these markers were polymorphic among the 40 coconut accessions. The most abundant class of SSRs identified from GBS sequences in this study was dinucleotide repeats. Similar results regarding abundant classes of coconut SSRs have been reported previously^42^. The most frequently identified type of dinucleotide repeat in the present study was AG/CT, similar to that identified for gene-based SRRs in a previous report^8^. However, this dinucleotide category was different from that identified from a whole-genome assembly, which was AT/TA^42^. Our newly developed SSR markers were tested for their effectiveness by using them to assess the genetic diversity of a collection of 40 coconut accessions. PIC values of these SSR markers ranged from 0.05 to 0.68. The majority of the SSR markers (64 markers) displayed low to moderate PIC values (PIC values < 0.50; Table 3). Low PIC values in these SSR markers due to low levels of polymorphism among the genotypes evaluated (Supplementary Information; Fig. S1). Gene diversity (He) based on the 74 SSR markers was observed to be higher in tall coconut accessions than in dwarf accessions, as the average gene diversity values in tall coconut and dwarf coconut accessions were 0.42 and 0.20, respectively. These values were lower than the overall mean gene diversity values previously reported for world coconut populations, which were 0.70 and 0.37 for tall and dwarf coconuts, respectively^12^. Similar to what was reflected by the observed gene diversity, the overall allelic richness of the dwarf accessions was lower than that of the tall accessions. The dwarf accessions included in the present study were less heterozygous; ten out of the 18 accessions were heterozygous at one to ten loci. However, there were three dwarf accessions (ACC.06 (Cameroon yellow dwarf), ACC.15 (Nok Khum), and ACC.44 (Khom)) that were substantially heterozygous. According to the PCoA analysis, these three dwarf accessions likely clustered together with the other five tall accessions, i.e., ACC.09 (Kalok), ACC.11 (Tahiti tall), ACC.26 (Thailand tall Ko Samui), ACC.24 (MaWa) and ACC.37 (Thailand tall Ko Chang). Moreover, according to the STRUCTURE results, the three dwarf accessions exhibited mixed genotypes from Cluster II (mainly tall accessions) and Cluster III (dwarf accessions). Therefore, it is possible that these three dwarf accessions with high heterozygosity were derived from outcrosses between dwarf and tall coconuts. It might also be the case that some other tall and dwarf coconut accessions of Thailand were cross-pollinated or crossbred between the two groups. It is interesting that a foreign dwarf accession, ACC.05 (Papua New Guinea brown dwarf), clustered together with other Thailand dwarf accessions in Cluster III. The SSR markers clearly divided the tall accessions into two groups as in Cluster I and Cluster II. This indicates that two classes of Thai tall coconut varieties derived from two different origins. In comparison, Cluster II included all Thai tall varieties in the Pacific region and was closer to Cluster III (dwarf accessions) than Cluster I, which included Thai tall varieties from Thailand’s Indian Ocean coast. This result confirms the previous findings^12^. Based on the analysis of 94 coconut varieties/populations, also comprising four tall and seven dwarf coconut varieties from Thailand, Perera et al. identified two main groups of coconut genotypes, one group comprising all the Talls from Southeast Asia, the Pacific, the west cost of Panama and all Dwarf, and another group comprising all Talls from south Asia, Africa, and the Indian Ocean coast. With more Thai coconut accessions and more SSR markers, our findings expanded previous knowledge on genetic diversity and coconut population structure in Thailand.

The method of SSR mining using GBS sequences applied in this study has proven to be efficient in coconut and could be applied for other tree plants with a large genome size or those without a complete genome reference. The newly developed and validated SSR markers could be useful for genetic diversity studies, which are an essential component of the characterization and utilization of coconut germplasms and are useful for the molecular breeding of coconuts.

## Materials and methods

### Plant materials

A total of 51 coconut accessions collected from plantations in different locations in Thailand were used in this study. Among these coconut accessions, 38, comprising 20 tall accessions and 18 dwarf accessions, were used to generate GBS sequence data. Forty coconut accessions were used to validate SSR markers. Among these accessions, 27 overlapped with those used in GBS sequencing. (Supplementary Information; Table S1).

### DNA extraction, GBS library preparation, and sequencing

Genomic DNA was isolated from 100 mg of young leaf tissue using the DNeasy Plant Mini Kit (Qiagen, USA) following the manufacturer’s instructions. The DNA of each sample was quantified using a NanoDrop 8000 spectrophotometer (Thermo Fisher Scientific, USA) and adjusted to 50 ng/μl. GBS libraries for each genotype were generated from 50 μl of genomic DNA using the restriction enzyme *Ape*KI following the GBS protocol as previously described^43^. GBS sequencing was performed using the Illumina HiSeq 2000 platform at the Beijing Genomics Institute (BGI, Shenzen, China).

### GBS data processing and microsatellite mining

The Illumina raw reads were preprocessed by simultaneously demultiplexing and removing low-quality reads using the *process_radtags* component of Stacks v1.39 software^28^. Reads that did not contain the restriction site of *Ape*KI were also discarded in the preprocessing step. The preprocessed clean reads were then analyzed using the core Stack pipeline containing the *ustacks* and *cstacks* components of Stacks v.1.39 software with default parameters and without using a reference genome. Each consensus sequence resulting from the Stack pipeline was then screened for simple sequence repeats (SSRs) using MISA with default parameters^29^. The acquired SSRs were considered to only represent those containing perfect repeats of SSRs whose basic motifs ranged from 2 to 6 bp with defined minimum repeat units of six iterations for dinucleotide repeats and five iterations for tri-, tetra-, penta-, and hexanucleotide repeats.

### Coconut SSR primer design

The BatchPrimer3 (version 2.3.7) program^31^ was employed to design oligonucleotide primers using the SSR flanking sequences. Putative SSR markers were selected based on the following parameters: primer length between 18–23 bp, PCR product length between 50 and 100 bp, primer melting temperature (Tm) between 52–60 °C with an optimum of 55 °C, and GC content of 30–67%. All putative markers were computationally validated across 38 genotypes to verify their polymorphism. Polymorphic SSR loci were defined as SSR polymorphisms present in the consensus sequences of the supporting accessions (at least 20 accessions).

### PCR validation of SSR markers

A total of 100 SSR primer pairs were selected for PCR screening in 40 diverse coconut accessions. PCR amplification was conducted in reaction mixtures with a total volume of 10 µl containing 2 µl of 20 ng/µl genomic DNA, 5X KAPA2G Buffer B with MgCl_2_, 5 U/μl of KAPA2G Robust HotStart DNA Polymerase (KAPA2G Robust HotStart PCR Kits, Kapa Biosystems, USA), 2 mM MgCl_2_, each SSR primer at 0.5 µM and 0.2 µM dNTP mix. Briefly, the PCR cycles consisted of initial denaturation at 95 °C for 3 min, followed by 35 cycles of denaturation at 95 °C for 30 s, primer annealing at a temperature depending on the primers for 30 s, and extension at 72 °C for 90 s, with a final extension at 72 °C for 10 min. The PCR products were electrophoretically separated on 4.5% denaturing polyacrylamide gels and visualized by silver staining. The genotypes characterized according to each marker were determined by allelic size differences in comparison to a 100 bp DNA ladder.

### SSR data analysis

We examined the genetic diversity of the 40 coconut accessions using the newly developed SSR markers. The individual bands amplified by the SSR primers in the SSR banding profile were scored as present (1) or absent (0). PowerMarker version 3.25^45^ was used to calculate polymorphic information content (PIC), the numbers of alleles, gene diversity, and the major allele frequency (MAF) and to construct the neighbor-joining phylogram using Nei’s distance dissimilarity matrix^46^. The STRUCTURE algorithm^47^ was run using a model with admixture and correlated allele frequencies, with 3 independent replicates run for each genetic cluster (K) value, with K ranging from 1 to 8, using a burn-in of 100,000 steps and a run length of 100,000 Markov Chain Monte Carlo (MCMC) iterations. Ln(PD) values were derived for each K and plotted to identify the plateau of ΔK^48^. The final population structure was calculated using the web-based software STRUCTURE HARVESTER version 0.6.92^49^. Individuals were placed into the respective subpopulation based on the highest percentage of membership (q). Principal coordinate analysis (PCoA) was performed using DARwin 6.0 software^33^ based on dissimilarity distances estimated between pairs of individuals. The exploratory Discriminant Analysis of Principal Components (DAPC) was applied using the *adegenet* package^34^ (function dapc). The analysis was performed without prior information on individual populations. The optimal number of clusters for assessing the best supported model was selected based on the Bayesian Information Criterion (BIC), as suggested by Jombart et al.^50^. The correlation between pairwise genetic distance and geographic distance among each Thai coconut accession was performed using a Mantel test^51^ as implemented in the GenAlEx 6.4^35^.

## Supplementary information

Supplementary Table S1

Supplementary Table S2

Supplementary Table S3

Supplementary Table S4

Supplementary Table S5

Supplementary Figure S1

Supplementary Figure S2

Supplementary Figure S3

Supplementary Figure S4

Supplementary Figure S5

## Data Availability

The GBS sequencing data used in this study can be freely and openly accessed at the NCBI Sequence Read Archive via the identifier PRJNA645608.
