# Supplementary material for: Mining and validation of novel genotyping-by-sequencing (GBS)-based simple sequence repeats (SSRs) and their application for the estimation of the genetic diversity and population structure of coconuts (Cocos nucifera L.) in Thailand
Source: Hortic Res. 2020 Oct 1;7:156. doi: 10.1038/s41438-020-00374-1 (PMC7527488; doi:10.1038/s41438-020-00374-1)
Supplement: Supplementary file 2 — Supplementary Table S2 [file 41438_2020_374_MOESM2_ESM.docx]

**Supplementary Table S2 List of primers for 100 selected SSR loci**

| **Index** | **Name** | **Seq (5'-3')** | **Length** | **Tm** | **GC%** | **Motif** | **Product size range (bp)** |
| --- | --- | --- | --- | --- | --- | --- | --- |
| 1 | CnSSR_1F | GCATGCACGAATAAGGATA | 19 | 55 | 42.11 | (TA)n | 90-92 |
|  | CnSSR_1R | CTGAACTGAGATTCAAGAGGA | 21 | 55 | 42.86 |  |  |
| 2 | CnSSR_2F | CCAACCAAGCCACCATTA | 18 | 58 | 50 | (CT)n | 82-90 |
|  | CnSSR_2R | TGACCTCGATTGTGAATTTTA | 21 | 56 | 33.33 |  |  |
| 3 | CnSSR_3F | TCTGGTCCTCAAAAGGTGT | 19 | 56 | 47.37 | (TC)n | 96-100 |
|  | CnSSR_3R | GAAGCTGGAACAGTAGAAACA | 21 | 55 | 42.86 |  |  |
| 4 | CnSSR_4F | CAGCAGGTGCAAATAGTTTT | 20 | 56 | 40 | (AG)n | 83-95 |
|  | CnSSR_4R | TTTGACAAAGTCTCACCAGAT | 21 | 55 | 38.1 |  |  |
| 5 | CnSSR_5F | AGAGGGAAAGAGAGATTATGG | 21 | 54 | 42.86 | (GA)n | 88-94 |
|  | CnSSR_5R | AAGCCTTGTAAACCAAAGG | 19 | 55 | 42.11 |  |  |
| 6 | CnSSR_6F | CAGCAACAGAGAAACAGGA | 19 | 55 | 47.37 | (AG)n | 94-96 |
|  | CnSSR_6R | TCTCTACCTCTCCCTCCTTC | 20 | 55 | 55 |  |  |
| 7 | CnSSR_7F | AGCATGAGGTGGTTCATCT | 19 | 55 | 47.37 | (CTC)n | 96-99 |
|  | CnSSR_7R | GGCATCGTGCCTGATGTG | 18 | 62 | 61.11 |  |  |
| 8 | CnSSR_8F | CCAATGTATTGTGAGATGGAG | 21 | 56 | 42.86 | (AG)n | 82-86 |
|  | CnSSR_8R | TTCTTCTTCTCTTTTCGCTCT | 21 | 55 | 38.1 |  |  |
| 9 | CnSSR_9F | CAGCAGAGTAGACCTATTTTAT | 22 | 50 | 36.36 | (AG)n | 100-114 |
|  | CnSSR_9R | GTGTGTGTTTCTTGGTCTACA | 21 | 54 | 42.86 |  |  |
| 10 | CnSSR_10F | GCTTTAGGTCTCCTCACTTCT | 21 | 55 | 47.62 | (TC)n | 80-84 |
|  | CnSSR_10R | ATGTAAATAAAACCCCCTGTG | 21 | 56 | 38.1 |  |  |
| 11 | CnSSR_11F | TCGTCGATCTACCATAAGAAA | 21 | 55 | 38.1 | (AC)n | 90-92 |
|  | CnSSR_11R | TTTGGAACTTGATCAGCATAC | 21 | 55 | 38.1 |  |  |
| 12 | CnSSR_12F | AGCAACATTTGAGGTTTATATG | 22 | 54 | 31.82 | (GA)n | 91-95 |
|  | CnSSR_12R | AACTCTCTTCCACCTTGTAGG | 21 | 55 | 47.62 |  |  |
| 13 | CnSSR_13F | TGGGCTCTGTTTCCGAAC | 18 | 60 | 55.56 | (GA)n | 80-90 |
|  | CnSSR_13R | CACCCTCCAATCCCTCTC | 18 | 58 | 61.11 |  |  |
| 14 | CnSSR_14F | AGCCTAGTCAAGGAAATAAGC | 21 | 55 | 42.86 | (AT)n | 82-84 |
|  | CnSSR_14R | AGGATTTATCTCTTTTGCATGT | 22 | 55 | 31.82 |  |  |
| 15 | CnSSR_15F | ACAAAGATCAAGTTGCAAAGA | 21 | 55 | 33.33 | (GA)n | 84-86 |
|  | CnSSR_15R | TCTTAATTATGCGATTACTCCTC | 23 | 55 | 34.78 |  |  |
| 16 | CnSSR_16F | CTTTGTTTTTCCCCTATTTGT | 21 | 55 | 33.33 | (TC)n | 84-94 |
|  | CnSSR_16R | ACTCTGGTGTAGGTGCAAAG | 20 | 55 | 50 |  |  |
| 17 | CnSSR_17F | AGCCATGCACCGTGGAAT | 18 | 63 | 55.56 | (CT)n | 91-111 |
|  | CnSSR_17R | AAGAAGAACTCCCAAACCAC | 20 | 56 | 45 |  |  |
| 18 | CnSSR_18F | AGCACATTCTCAGAAGAAAAA | 21 | 54 | 33.33 | (TG)n | 99-101 |
|  | CnSSR_18R | GCACAAGGATATGAATAACA | 20 | 51 | 35 |  |  |
| 19 | CnSSR_19F | AGGGGCGTGGCTGTAGGT | 18 | 63 | 66.67 | (GGT)n | 90-93 |
|  | CnSSR_19R | ACGAACCCGCACCCTACC | 18 | 63 | 66.67 |  |  |
| 20 | CnSSR_20F | AAACTGGTGGGAGGTGTG | 18 | 57 | 55.56 | (TC)n | 90-94 |
|  | CnSSR_20R | AAATTAAAGGAAGTCTCAGCAC | 22 | 55 | 36.36 |  |  |
| 21 | CnSSR_21F | GCCTTAATGATCTCAACCTTAC | 22 | 55 | 40.91 | (AT)n | 92-94 |
|  | CnSSR_21R | CCTAACCTGCACTCTTGGA | 19 | 56 | 52.63 |  |  |
| 22 | CnSSR_22F | TCTTGTACCTATGCCACCTTA | 21 | 55 | 42.86 | (CT)n | 91-99 |
|  | CnSSR_22R | TATAAAACAGGAGCGGGTCTA | 21 | 57 | 42.86 |  |  |
| 23 | CnSSR_23F | TGATGTTTAAGGTTTGGTGTT | 21 | 55 | 33.33 | (CT)n | 92-96 |
|  | CnSSR_23R | ACCTATTTCGTTCTTACCTATT | 22 | 51 | 31.82 |  |  |
| 24 | CnSSR_24F | AGCTAAATTAGAACGGTCCTG | 21 | 55 | 42.86 | (TA)n | 88-90 |
|  | CnSSR_24R | CCAGTCTTCTATTCAACATCCT | 22 | 55 | 40.91 |  |  |
| 25 | CnSSR_25F | CTTTACTTAGCTGTGGAGCAA | 21 | 55 | 42.86 | (AT)n | 96-100 |
|  | CnSSR_25R | GATTGCTGTTTAGGTTTCG | 19 | 53 | 42.11 |  |  |
| 26 | CnSSR_26F | CCTGCAACAGAAGCAATC | 18 | 55 | 50 | (AAT)n | 90-93 |
|  | CnSSR_26R | GATGGGATTCGTTTGAAAT | 19 | 55 | 36.84 |  |  |
| 27 | CnSSR_27F | AGGGGCGTGGCTGTAGGT | 18 | 63 | 66.67 | (GGT)n | 90-93 |
|  | CnSSR_27R | ACGAACCCGCACCCTACC | 18 | 63 | 66.67 |  |  |
| 28 | CnSSR_28F | GGAGCTCTCACAAGTCAAATA | 21 | 55 | 42.86 | (AG)n | 87-99 |
|  | CnSSR_28R | GGTCCCATTTCTTCTTCTCTA | 21 | 55 | 42.86 |  |  |
| 29 | CnSSR_29F | CGTTCAACGAGGCAGGTT | 18 | 60 | 55.56 | (GA)n | 87-89 |
|  | CnSSR_29R | CTTTCCCTATTGGCAGTATTT | 21 | 55 | 38.1 |  |  |
| 30 | CnSSR_30F | TGATGCTCTCAATGATCTCTT | 21 | 55 | 38.1 | (TA)n | 80-82 |
|  | CnSSR_30R | CATCTTAAAAGGGTAATATAGCA | 23 | 53 | 30.43 |  |  |
| 31 | CnSSR_31F | CACCAGCAATTGAGACTCTAC | 21 | 55 | 47.62 | (ACC)n | 81-84 |
|  | CnSSR_31R | CAACGATGATGAGGAAGC | 18 | 55 | 50 |  |  |
| 32 | CnSSR_32F | AAGGGGCTTTGATGTAATAAT | 21 | 55 | 33.33 | (TA)n | 87-95 |
|  | CnSSR_32R | TATGGTAGGCTTTCTTTTTCC | 21 | 55 | 38.1 |  |  |
| 33 | CnSSR_33F | GAACCCACCAAAAAGAGAG | 19 | 55 | 47.37 | (AG)n | 95-111 |
|  | CnSSR_33R | TCCTTGCTGTACTACTTGCTC | 21 | 55 | 47.62 |  |  |
| 34 | CnSSR_34F | AAAACGCCAAAACCATTA | 18 | 54 | 33.33 | (TTGT)n | 83-87 |
|  | CnSSR_34R | TTGAAAGAAGCAGAAGAAGAA | 21 | 55 | 33.33 |  |  |
| 35 | CnSSR_35F | GGATCGGGCTGATCTTAT | 18 | 55 | 50 | (TA)n | 89-95 |
|  | CnSSR_35R | CGGATGAAGGCATGTATATTA | 21 | 55 | 38.1 |  |  |
| 36 | CnSSR_36F | CCCTAGCATTCAAACATACAT | 21 | 54 | 38.1 | (CT)n | 87-103 |
|  | CnSSR_36R | CGAGACAAATCGTACCCATA | 20 | 56 | 45 |  |  |
| 37 | CnSSR_37F | AGAGGGTTTGATGGAATAAAT | 21 | 54 | 33.33 | (CAAAG)n | 80-85 |
|  | CnSSR_37R | AGGTATGGTCAGTCATTTTTG | 21 | 54 | 38.1 |  |  |
| 38 | CnSSR_38F | CATGTACCTGCTCTCATTCAT | 21 | 55 | 42.86 | (TGT)n | 88-91 |
|  | CnSSR_38R | CTATCAGAACCATCCAACATC | 21 | 55 | 42.86 |  |  |
| 39 | CnSSR_39F | CCATTCTAGATATCCCACACC | 21 | 56 | 47.62 | (CT)n | 82-92 |
|  | CnSSR_39R | TCTTCTCAGATTTGTGCCTAA | 21 | 55 | 38.1 |  |  |
| 40 | CnSSR_40F | GCCAGCACAAGGGATATT | 18 | 56 | 50 | (CGG)n | 91-94 |
|  | CnSSR_40R | GGAAAAGAGGATGAAGAAGAG | 21 | 55 | 42.86 |  |  |
| 41 | CnSSR_41F | CATAGAAGTTGGTCGAAAATG | 21 | 55 | 38.1 | (TA)n | 97-101 |
|  | CnSSR_41R | AGCTATTGGAACCACTCCA | 19 | 56 | 47.37 |  |  |
| 42 | CnSSR_42F | CCAGAGTTTTCGTTTTGTTTT | 21 | 56 | 33.33 | (TG)n | 86-90 |
|  | CnSSR_42R | TTTGAACAGCCACACTCC | 18 | 55 | 50 |  |  |
| 43 | CnSSR_43F | ATGCAGAGAAGCTCTATTGTG | 21 | 55 | 42.86 | (CT)n | 90-94 |
|  | CnSSR_43R | TTTAAGCGATCCTCCAGTC | 19 | 55 | 47.37 |  |  |
| 44 | CnSSR_44F | CTAAGCGCTAAGATGATGAGA | 21 | 55 | 42.86 | (AG)n | 80-82 |
|  | CnSSR_44R | ATCGCCATCTCTCTCTCC | 18 | 55 | 55.56 |  |  |
| 45 | CnSSR_45F | GAGTGGGGTCCGTCTAGC | 18 | 58 | 66.67 | (GA)n | 85-91 |
|  | CnSSR_45R | GTGAGCTCAGAGGAGTTGTG | 20 | 56 | 55 |  |  |
| 46 | CnSSR_46F | TATCCAATCTCACCCCATT | 19 | 55 | 42.11 | (CT)n | 86-92 |
|  | CnSSR_46R | CTCTCTCATGAACGCAGAGT | 20 | 56 | 50 |  |  |
| 47 | CnSSR_47F | CCCGGGTAAGCTATAAGTCT | 20 | 55 | 50 | (TC)n | 86-96 |
|  | CnSSR_47R | GTGAGGGAGGACGAAAAG | 18 | 56 | 55.56 |  |  |
| 48 | CnSSR_48F | ATCACAATGCCTTTTGTACC | 20 | 55 | 40 | (TG)n | 85-101 |
|  | CnSSR_48R | TGGTTGAACTTAACTGTCTTCA | 22 | 55 | 36.36 |  |  |
| 49 | CnSSR_49F | CAGCCCTCTGATAGTCACC | 19 | 56 | 57.89 | (TC)n | 85-89 |
|  | CnSSR_49R | ACTGACATTGCAGAGAGAGAA | 21 | 55 | 42.86 |  |  |
| 50 | CnSSR_50F | AAATTACTGGATCCCCTACC | 20 | 55 | 45 | (AC)n | 88-90 |
|  | CnSSR_50R | AAGCCCTATCATCTTAACCTT | 21 | 54 | 38.1 |  |  |
| 51 | CnSSR_51F | TCAACCCTCAAAGTGATTCTA | 21 | 55 | 38.1 | (AT)n | 82-88 |
|  | CnSSR_51R | AAGGAAGAAAATCTGCATGAC | 21 | 56 | 38.1 |  |  |
| 52 | CnSSR_52F | ATGGTGCTCTCCCTCGAC | 18 | 59 | 61.11 | (TC)n | 96-102 |
|  | CnSSR_52R | GCTAACTCTTCCTTCGAAACT | 21 | 55 | 42.86 |  |  |
| 53 | CnSSR_53F | CCATTTCTCTTGTCAACCTAC | 21 | 54 | 42.86 | (TTC)n | 80-95 |
|  | CnSSR_53R | ATCAAAAGACCTATGCACAAA | 21 | 55 | 33.33 |  |  |
| 54 | CnSSR_54F | AACCATGGGCTCTCGACT | 18 | 58 | 55.56 | (AT)n | 81-89 |
|  | CnSSR_54R | ATGACGCAAGGAAAGCTC | 18 | 56 | 50 |  |  |
| 55 | CnSSR_55F | AGCCAGTGTTTTCAGTGC | 18 | 55 | 50 | (GA)n | 80-90 |
|  | CnSSR_55R | CCTCCCTCACTCTGTTTCTT | 20 | 56 | 50 |  |  |
| 56 | CnSSR_56F | ATCGCACTCTTCCTCTCC | 18 | 55 | 55.56 | (TC)n | 87-89 |
|  | CnSSR_56R | GAGAAAACATGGGGCAAG | 18 | 57 | 50 |  |  |
| 57 | CnSSR_57F | CCGTCGTCAGTACCAAATTAT | 21 | 57 | 42.86 | (CT)n | 80-82 |
|  | CnSSR_57R | GTAGTCCCCAAGGAAGAGAG | 20 | 55 | 55 |  |  |
| 58 | CnSSR_58F | CCTGGAATCAACCATAATCTA | 21 | 54 | 38.1 | (GA)n | 91-97 |
|  | CnSSR_58R | TAAGCATGTTAATGCTCTCCT | 21 | 54 | 38.1 |  |  |
| 59 | CnSSR_59F | ATGGAAACCATGGCAGAC | 18 | 57 | 50 | (GTG)n | 80-83 |
|  | CnSSR_59R | TATGGACATTGGACATCCTTA | 21 | 55 | 38.1 |  |  |
| 60 | CnSSR_60F | GCAGTCCATCAAATAAAAAGA | 21 | 55 | 33.33 | (AG)n | 88-90 |
|  | CnSSR_60R | GAGTGAGCATCCAATCCTT | 19 | 55 | 47.37 |  |  |
| 61 | CnSSR_61F | AAATCGTTGGAGTGCAAA | 18 | 55 | 38.89 | (CTT)n | 83-85 |
|  | CnSSR_61R | AATGCTAACCCAACCTGATA | 20 | 55 | 40 |  |  |
| 62 | CnSSR_62F | CTGGGATCCTCAGTTGTTAAT | 21 | 56 | 42.86 | (CT)n | 80-86 |
|  | CnSSR_62R | AAGAAGATGACAAAGATTAGGT | 22 | 52 | 31.82 |  |  |
| 63 | CnSSR_63F | GCAGCAGGAAGCAAATAATA | 20 | 56 | 40 | (GTTTG)n | 84-89 |
|  | CnSSR_63R | CCTTCTTGAGCTTAGAGAAAAA | 22 | 55 | 36.36 |  |  |
| 64 | CnSSR_64F | TCGTAATAAAAAGGAGTACCG | 21 | 54 | 38.1 | (AG)n | 82-84 |
|  | CnSSR_64R | TTTCTTACTAGATGGGTCACG | 21 | 55 | 42.86 |  |  |
| 65 | CnSSR_65F | TGCAGAGATAGGAAGAGATAGAG | 23 | 55 | 43.48 | (AG)n | 82-86 |
|  | CnSSR_65R | CAACCAGAGGAGAGCAGAG | 19 | 56 | 57.89 |  |  |
| 66 | CnSSR_66F | AGGTCTCAAGGCCGACCAC | 19 | 63 | 63.16 | (TCC)n | 80-89 |
|  | CnSSR_66R | GACGGTGACACGGTGAGT | 18 | 58 | 61.11 |  |  |
| 67 | CnSSR_67F | CGACTTCCCTAGTTCTTTTTC | 21 | 55 | 42.86 | (AT)n | 82-86 |
|  | CnSSR_67R | CTTTCTTTGTTTATGCTGGAA | 21 | 55 | 33.33 |  |  |
| 68 | CnSSR_68F | AGCACTTGAGATCAAAATGAA | 21 | 55 | 33.33 | (AT)n | 87-89 |
|  | CnSSR_68R | TACGTACACCACCTTTGATTC | 21 | 55 | 42.86 |  |  |
| 69 | CnSSR_69F | ATTGCCGAGGCCGGTGGA | 18 | 69 | 66.67 | (GGA)n | 93-96 |
|  | CnSSR_69R | AAGGTGAGGGAGAAGAAGAG | 20 | 55 | 50 |  |  |
| 70 | CnSSR_70F | GTTGATCTCTCCTTTGCTTTT | 21 | 55 | 38.1 | (TC)n | 81-83 |
|  | CnSSR_70R | ACAGCCCCTATGTAGACTAGC | 21 | 55 | 52.38 |  |  |
| 71 | CnSSR_71F | CCTTTAGAGGTCGTCTCTCC | 20 | 56 | 55 | (CT)n | 91-93 |
|  | CnSSR_71R | GACACAATAGAGAGGGCAGA | 20 | 55 | 50 |  |  |
| 72 | CnSSR_72F | TGAGTTTAACAGGGTGGTTAC | 21 | 54 | 42.86 | (GAA)n | 92-98 |
|  | CnSSR_72R | GAGACAAGGCAGTCATCATAG | 21 | 55 | 47.62 |  |  |
| 73 | CnSSR_73F | CAGCTGGAGACAAGAATTAAG | 21 | 54 | 42.86 | (AG)n | 95-101 |
|  | CnSSR_73R | GGATCCTCAGTTGTTAATGG | 20 | 55 | 45 |  |  |
| 74 | CnSSR_74F | AGATTACGGATGGGAAGAGT | 20 | 55 | 45 | (TTA)n | 81-84 |
|  | CnSSR_74R | GAAACAAAACCACTAGTCATAAA | 23 | 53 | 30.43 |  |  |
| 75 | CnSSR_75F | TGATGTTTAAGGTTTGGTGTT | 21 | 55 | 33.33 | (CT)n | 90-94 |
|  | CnSSR_75R | ACCTATTTCGTTCTTACCTATT | 22 | 51 | 31.82 |  |  |
| 76 | CnSSR_76F | GTCGTTCACCAGGACATC | 18 | 54 | 55.56 | (CT)n | 85-87 |
|  | CnSSR_76R | CTTCGCCGCTTTGGACTT | 18 | 61 | 55.56 |  |  |
| 77 | CnSSR_77F | ATTTTAGCTTTCTTGGATTCG | 21 | 55 | 33.33 | (TC)n | 83-85 |
|  | CnSSR_77R | GCAAGCATCAGATGTTATAG | 20 | 51 | 40 |  |  |
| 78 | CnSSR_78F | AGCCCTCCAACATCCTTG | 18 | 59 | 55.56 | (TC)n | 97-101 |
|  | CnSSR_78R | GAGAAAGAAGCAAAGAGAGAAA | 22 | 55 | 36.36 |  |  |
| 79 | CnSSR_79F | ACCTTGGATTTACAGCCTAAC | 21 | 55 | 42.86 | (TC)n | 84-86 |
|  | CnSSR_79R | AAAGAGCAGAAACACTGGATA | 21 | 54 | 38.1 |  |  |
| 80 | CnSSR_80F | TGCTGTTGTTACTATTTCGATG | 22 | 56 | 36.36 | (GAG)n | 87-90 |
|  | CnSSR_80R | TCATCCTCGAGGTCCTTAC | 19 | 55 | 52.63 |  |  |
| 81 | CnSSR_81F | ATGCTACAATCACCAATTCAC | 21 | 55 | 38.1 | (GT)n | 85-87 |
|  | CnSSR_81R | TCCCTTAGAGTGAACAGTTTG | 21 | 55 | 42.86 |  |  |
| 82 | CnSSR_82F | CAGCGCCATAGGTTTATATG | 20 | 56 | 45 | (TG)n | 99-101 |
|  | CnSSR_82R | GAGCGGGATTTATGCAAT | 18 | 56 | 44.44 |  |  |
| 83 | CnSSR_83F | TTTTCTCTACGTGCTGAAGG | 20 | 56 | 45 | (TAA)n | 84-86 |
|  | CnSSR_83R | CGATGCTGGGCCGGGATA | 18 | 68 | 66.67 |  |  |
| 84 | CnSSR_84F | TTATTATGATAGCGTGCACAT | 21 | 54 | 33.33 | (TG)n | 85-87 |
|  | CnSSR_84R | ATTTTCAAACATGGGTACAT | 20 | 52 | 30 |  |  |
| 85 | CnSSR_85F | CTTTGGACAAAATGCATGA | 19 | 56 | 36.84 | (CT)n | 84-98 |
|  | CnSSR_85R | AATTATCCACACACACACACA | 21 | 55 | 38.1 |  |  |
| 86 | CnSSR_86F | CTTCTTGTCCCTCTTTCACTC | 21 | 56 | 47.62 | (TC)n | 83-85 |
|  | CnSSR_86R | TAAAGGAATGCACCATCAAT | 20 | 56 | 35 |  |  |
| 87 | CnSSR_87F | TAGGTGCACAAGAATGTGAAT | 21 | 56 | 38.1 | (AAAG)n | 93-109 |
|  | CnSSR_87R | TTCTCATGTATTGTTTTCCTTCT | 23 | 55 | 30.43 |  |  |
| 88 | CnSSR_88F | CTGCTGAAAGTAATAATAACAAG | 23 | 51 | 30.43 | (AAC)n | 74-83 |
|  | CnSSR_88R | CAAGCCCAACTATGGTAAGTA | 21 | 55 | 42.86 |  |  |
| 89 | CnSSR_89F | CATCAGCCACCTGAAAAA | 18 | 56 | 44.44 | (GAA)n | 93-96 |
|  | CnSSR_89R | CATTAAATAGTCGGCTCCATC | 21 | 56 | 42.86 |  |  |
| 90 | CnSSR_90F | GCACTTGGTACCTTCAAATAA | 21 | 55 | 38.1 | (AG)n | 85-91 |
|  | CnSSR_90R | ATCACATAAATGCCAATTCAC | 21 | 55 | 33.33 |  |  |
| 91 | CnSSR_91F | TTTTTGTTCTTCTCATCCTTCT | 22 | 55 | 31.82 | (CTT)n | 80-83 |
|  | CnSSR_91R | TTTCTTCTTCTTCTCCTCCTT | 21 | 54 | 38.1 |  |  |
| 92 | CnSSR_92F | GCAGAGAAAGCACCATCTAAT | 21 | 56 | 42.86 | (AG)n | 98-104 |
|  | CnSSR_92R | TTGTCCTGTACGTTCTCTCTT | 21 | 54 | 42.86 |  |  |
| 93 | CnSSR_93F | AAGAGGATGGTAGGCATAAAC | 21 | 55 | 42.86 | (TA)n | 81-87 |
|  | CnSSR_93R | GCATACACTTGCTGTTGTCTA | 21 | 54 | 42.86 |  |  |
| 94 | CnSSR_94F | AAGAGCTCTAGATCTGGCAAT | 21 | 55 | 42.86 | (GA)n | 90-92 |
|  | CnSSR_94R | CTCCTTATTGATGGCCTTT | 19 | 54 | 42.11 |  |  |
| 95 | CnSSR_95F | CAATTTGCCTCCCTTAAAT | 19 | 54 | 36.84 | (AG)n | 91-95 |
|  | CnSSR_95R | TGCACCAACATAATTTACCA | 20 | 55 | 35 |  |  |
| 96 | CnSSR_96F | CATGGCATATCCAATATGTTT | 21 | 55 | 33.33 | (GT)n | 80-84 |
|  | CnSSR_96R | AGGAGTAACATGCATTTCTGT | 21 | 54 | 38.1 |  |  |
| 97 | CnSSR_97F | CAAAGCCACCATCCCTTC | 18 | 59 | 55.56 | (CGC)n | 87-90 |
|  | CnSSR_97R | CTACCGCTAGGCGACGAGGAG | 21 | 66 | 66.67 |  |  |
| 98 | CnSSR_98F | GGGCCAACCAATATAGCTC | 19 | 57 | 52.63 | (GGCTCA)n | 90-96 |
|  | CnSSR_98R | GGCTTAGGCGTCAATTTT | 18 | 55 | 44.44 |  |  |
| 99 | CnSSR_99F | ACGGAGGGGCAAATGGAC | 18 | 63 | 61.11 | (GGA)n | 83-86 |
|  | CnSSR_99R | CCCGCCACCATCTCCTCT | 18 | 64 | 66.67 |  |  |
| 100 | CnSSR_100F | CATCATCCTCTCTTTTCCTTC | 21 | 56 | 42.86 | (CT)n | 96-98 |
|  | CnSSR_100R | GATTCGGCCTTTCAAATC | 18 | 56 | 44.44 |  |  |
