# Supplementary material for: Mining and validation of novel genotyping-by-sequencing (GBS)-based simple sequence repeats (SSRs) and their application for the estimation of the genetic diversity and population structure of coconuts (Cocos nucifera L.) in Thailand
Source: Hortic Res. 2020 Oct 1;7:156. doi: 10.1038/s41438-020-00374-1 (PMC7527488; doi:10.1038/s41438-020-00374-1)
Supplement: Supplementary file 3 — Supplementary Table S3 [file 41438_2020_374_MOESM3_ESM.docx]

**Supplementary Table S3** List of coconut accessions used in the study

| **Accession Code** | **Accession Name** | **GBS** | **marker validation** | **Geographic/Origin** | **Morphology** | **Fruit color** | **Remarks** |
| --- | --- | --- | --- | --- | --- | --- | --- |
| ACC.01 | Maphraeo #1 | ✓ |  | Thailand | Tall | Green | Spicata |
| ACC.02 | Nam Wan #1 | ✓ | ✓ | Thailand | Dwarf | Green | Sweet water |
| ACC.03 | Thung Kled | ✓ | ✓ | Thailand | Dwarf | Green |  |
| ACC.04 | Pak Chok #1 | ✓ | ✓ | Thailand | Tall | Green | Niu kafa |
| ACC.05 | Papua New Guinea brown dwarf | ✓ | ✓ | Papua New Guinea | Dwarf | Brown |  |
| ACC.06 | Cameroon yellow dwarf | ✓ | ✓ | Cameroon | Dwarf | Yellow |  |
| ACC.07 | Rennell Island Tall | ✓ |  | Solomon island | Tall | Green |  |
| ACC.08 | West African Tall | ✓ | ✓ | Ivory Coast | Tall | Green |  |
| ACC.09 | Kalok | ✓ | ✓ | Thailand | Tall | Green |  |
| ACC.10 | Thalai Roi | ✓ | ✓ | Thailand | Tall | Green | Many fruits per panicle |
| ACC.11 | Tahiti Tall | ✓ | ✓ | Tahiti | Tall | Green |  |
| ACC.12 | Pak Chok #2 | ✓ | ✓ | Island, Thailand | Tall | Green | Niu kafa; Long fruits |
| ACC.13 | Mu Si Som | ✓ | ✓ | Thailand | Dwarf | Orange |  |
| ACC.14 | Nam Hom #1 | ✓ |  | Thailand | Dwarf | Green |  |
| ACC.15 | Nok Khum | ✓ | ✓ | Thailand | Dwarf | Green |  |
| ACC.16 | Nali-ke | ✓ | ✓ | Thailand | Dwarf | Green |  |
| ACC.17 | Thailand Tall Nakhon Si Thammarat | ✓ | ✓ | Thailand | Tall | Brown |  |
| ACC.18 | Thailand Tall Thap Sakae | ✓ | ✓ | Thailand | Tall | Green |  |
| ACC.19 | Sri Lanka Tall | ✓ | ✓ | Sri Lanka | Tall | Green |  |
| ACC.20 | Thailand Tall Sawi #1 | ✓ | ✓ | Thailand | Tall | Brown |  |
| ACC.21 | Thailand Tall Sawi #2 | ✓ | ✓ | Thailand | Tall | Green |  |
| ACC.22 | Thailand Tall Sawi #3 | ✓ | ✓ | Thailand | Tall | Green |  |
| ACC.23 | Mu Si Luang | ✓ |  | Thailand | Dwarf | Yellow |  |
| ACC.24 | MaWa | ✓ | ✓ | Thailand | Tall | Brown | Hybrid (MYD x WAT) |
| ACC.25 | King coconut | ✓ |  | Sri Lanka | Dwarf | Orange |  |
| ACC.26 | Thailand Tall Ko Samui | ✓ | ✓ | Island, Thailand | Tall | Green |  |
| ACC.27 | Thailand Tall Ko Pha-ngan | ✓ | ✓ | Island, Thailand | Tall | Green |  |
| ACC.28 | NDK | ✓ |  | Thailand | Dwarf | Green | Makapuno hybrid |
| ACC.29 | YDK | ✓ |  | Thailand | Dwarf | Yellow | Makapuno hybrid |
| ACC.31 | Mu Si Nu | ✓ |  | Thailand | Dwarf | Green |  |
| ACC.32 | Maphrao So #1 | ✓ |  | Thailand | Tall | Green |  |
| ACC.33 | Ratchaburi 2 | ✓ | ✓ | Thailand | Dwarf | Green |  |
| ACC.34 | Ratchaburi 3 | ✓ | ✓ | Thailand | Dwarf | Green |  |
| ACC.35 | Ratchaburi 1 | ✓ | ✓ | Thailand | Dwarf | Green |  |
| ACC.36 | Nam Wan #2 |  | ✓ | Thailand | Dwarf | Green |  |
| ACC.37 | Thailand Tall Ko Chang | ✓ | ✓ | Island, Thailand | Tall | Green |  |
| ACC.38 | Maphrao Tuen Dok | ✓ |  | Thailand | Dwarf | Brown |  |
| ACC.39 | Maphraeo #2 | ✓ |  | Thailand | Tall | Green | Spicata |
| ACC.40 | Nam Hom #2 | ✓ | ✓ | Thailand | Dwarf | Green |  |
| ACC.41 | Pathiu |  | ✓ | Thailand | Dwarf | Green |  |
| ACC.42 | Nam Wan #3 |  | ✓ | Thailand | Dwarf | Green |  |
| ACC.43 | Nam Hom Kathi |  | ✓ | Thailand | Dwarf | Green | Aromatic makapuno |
| ACC.44 | Khom |  | ✓ | Thailand | Dwarf | Green |  |
| ACC.45 | Maphrao So #2 |  | ✓ | Thailand | Tall | Green |  |
| ACC.46 | Tha Nan |  | ✓ | Thailand | Tall | Green |  |
| ACC.47 | Nam Hom #3 |  | ✓ | Thailand | Dwarf | Green |  |
| ACC.48 | Maphrao Fai Kathi |  | ✓ | Thailand | Tall | Green | Makapuno |
| ACC.49 | Nam Wan #3 |  | ✓ | Thailand | Dwarf | Green |  |
| ACC.50 | Mu Si Mo |  | ✓ | Thailand | Tall | Green |  |
| ACC.51 | Maphrao Fai |  | ✓ | Thailand | Tall | Red |  |
| ACC.52 | Phuang Roi Si Thong |  | ✓ | Thailand | Tall | Yellow |  |
