# Supplementary material for: Mining and validation of novel genotyping-by-sequencing (GBS)-based simple sequence repeats (SSRs) and their application for the estimation of the genetic diversity and population structure of coconuts (Cocos nucifera L.) in Thailand
Source: Hortic Res. 2020 Oct 1;7:156. doi: 10.1038/s41438-020-00374-1 (PMC7527488; doi:10.1038/s41438-020-00374-1)
Supplement: Supplementary file 4 — Supplementary Table S4 [file 41438_2020_374_MOESM4_ESM.docx]

**Table S4 Levels of heterozygosity in 40 coconut accessions determined by using 74 SSR markers**

| Accession | Name | Type | Heterozygous loci^a^ | missing^b^ | Availability^c^ | Heterozygosity^d^ |
| --- | --- | --- | --- | --- | --- | --- |
| ACC.02 | Nam Wan #1 | Dwarf | 6 | 0 | 74 | 8.11 |
| ACC.03 | Thung Kled | Dwarf | 3 | 0 | 74 | 4.05 |
| ACC.04 | Pak Chok #1 | Tall | 23 | 2 | 72 | 31.94 |
| ACC.05 | Papua New Guinea brown dwarf | Dwarf | 3 | 0 | 74 | 4.05 |
| ACC.06 | Cameroon yellow dwarf | Dwarf | 15 | 0 | 74 | 20.27 |
| ACC.08 | West African Tall | Tall | 25 | 3 | 71 | 35.21 |
| ACC.09 | Kalok | Tall | 13 | 0 | 74 | 17.57 |
| ACC.10 | Thalai Roi | Tall | 16 | 1 | 73 | 21.92 |
| ACC.11 | Tahiti Tall | Tall | 12 | 0 | 74 | 16.22 |
| ACC.12 | Pak Chok #2 | Tall | 26 | 1 | 73 | 35.62 |
| ACC.13 | Mu Si Som | Dwarf | 2 | 0 | 74 | 2.70 |
| ACC.15 | Nok Khum | Dwarf | 19 | 1 | 73 | 26.03 |
| ACC.16 | Nali-ke | Dwarf | 1 | 1 | 73 | 1.37 |
| ACC.17 | Thailand Tall Nakhon Si Thammarat | Tall | 23 | 0 | 74 | 31.08 |
| ACC.18 | Thailand Tall Thap Sakae | Tall | 16 | 2 | 72 | 22.22 |
| ACC.19 | Sri Lanka Tall | Tall | 30 | 2 | 72 | 41.67 |
| ACC.20 | Thailand Tall Sawi #1 | Tall | 25 | 0 | 74 | 33.78 |
| ACC.21 | Thailand Tall Sawi #2 | Tall | 11 | 1 | 73 | 15.07 |
| ACC.22 | Thailand Tall Sawi #3 | Tall | 15 | 0 | 74 | 20.27 |
| ACC.24 | MaWa | Tall | 35 | 1 | 73 | 47.95 |
| ACC.26 | Thailand Tall Ko Samui | Tall | 15 | 0 | 74 | 20.27 |
| ACC.27 | Thailand Tall Ko Pha-ngan | Tall | 17 | 0 | 74 | 22.97 |
| ACC.33 | Ratchaburi 2 | Dwarf | 10 | 0 | 74 | 13.51 |
| ACC.34 | Ratchaburi 3 | Dwarf | 1 | 0 | 74 | 1.35 |
| ACC.35 | Ratchaburi 1 | Dwarf | 1 | 1 | 73 | 1.37 |
| ACC.36 | Nam Wan #2 | Dwarf | 7 | 2 | 72 | 9.72 |
| ACC.37 | Thailand Tall Ko Chang | Tall | 17 | 0 | 74 | 22.97 |
| ACC.40 | Nam Hom #2 | Dwarf | 2 | 0 | 74 | 2.70 |
| ACC.41 | Pathiu | Dwarf | 3 | 0 | 74 | 4.05 |
| ACC.42 | Nam Wan #3 | Dwarf | 3 | 0 | 74 | 4.05 |
| ACC.43 | Nam Hom Kathi | Dwarf | 13 | 1 | 73 | 17.81 |
| ACC.44 | Khom | Dwarf | 23 | 0 | 74 | 31.08 |
| ACC.45 | Maphrao So #2 | Tall | 17 | 2 | 72 | 23.61 |
| ACC.46 | Tha Nan | Tall | 16 | 1 | 73 | 21.92 |
| ACC.47 | Nam Hom #3 | Dwarf | 5 | 1 | 73 | 6.85 |
| ACC.48 | Maphrao Fai Kathi | Tall | 14 | 2 | 72 | 19.44 |
| ACC.49 | Nam Wan #3 | Dwarf | 9 | 0 | 74 | 12.16 |
| ACC.50 | Mu Si Mo | Tall | 21 | 0 | 74 | 28.38 |
| ACC.51 | Maphrao Fai | Tall | 18 | 3 | 71 | 25.35 |
| ACC.52 | Phuang Roi Si Thong | Tall | 12 | 1 | 73 | 16.44 |

Remarks: a = number of heterozygous genotypes, b = number of missing data, c = number of available markers, d = percentage of heterozygosity (a/c * 100)
