# Supplementary material for: Mining and validation of novel genotyping-by-sequencing (GBS)-based simple sequence repeats (SSRs) and their application for the estimation of the genetic diversity and population structure of coconuts (Cocos nucifera L.) in Thailand
Source: Hortic Res. 2020 Oct 1;7:156. doi: 10.1038/s41438-020-00374-1 (PMC7527488; doi:10.1038/s41438-020-00374-1)
Supplement: Supplementary file 5 — Supplementary Table S5 [file 41438_2020_374_MOESM5_ESM.docx]

**Supplementary Table S5** 49 SSR markers subset of 74 SSR markers

| No. | **Marker** | **MAF** | **Number of observations** | **Number of alleles** | **He** | **Ho** | **PIC** |
| --- | --- | --- | --- | --- | --- | --- | --- |
| 1 | CnSSR03 | 0.77 | 39 | 4 | 0.37 | 0.26 | 0.32 |
| 2 | CnSSR04 | 0.85 | 39 | 3 | 0.27 | 0.13 | 0.25 |
| 3 | CnSSR05 | 0.54 | 40 | 5 | 0.65 | 0.35 | 0.61 |
| 4 | CnSSR06 | 0.59 | 38 | 2 | 0.48 | 0.71 | 0.37 |
| 5 | CnSSR09 | 0.49 | 40 | 9 | 0.64 | 0.05 | 0.57 |
| 6 | CnSSR10 | 0.81 | 40 | 2 | 0.30 | 0.08 | 0.26 |
| 7 | CnSSR12 | 0.65 | 40 | 2 | 0.46 | 0.30 | 0.35 |
| 8 | CnSSR13 | 0.84 | 40 | 3 | 0.28 | 0.13 | 0.26 |
| 9 | CnSSR16 | 0.33 | 39 | 6 | 0.73 | 0.41 | 0.68 |
| 10 | CnSSR17 | 0.79 | 39 | 2 | 0.33 | 0.10 | 0.27 |
| 11 | CnSSR20 | 0.60 | 40 | 2 | 0.48 | 0.20 | 0.36 |
| 12 | CnSSR21 | 0.79 | 40 | 3 | 0.34 | 0.23 | 0.29 |
| 13 | CnSSR22 | 0.83 | 40 | 4 | 0.30 | 0.18 | 0.28 |
| 14 | CnSSR23 | 0.78 | 40 | 3 | 0.37 | 0.38 | 0.33 |
| 15 | CnSSR25 | 0.71 | 38 | 4 | 0.46 | 0.26 | 0.43 |
| 16 | CnSSR28 | 0.46 | 40 | 7 | 0.69 | 0.43 | 0.64 |
| 17 | CnSSR31 | 0.50 | 40 | 2 | 0.50 | 0.25 | 0.38 |
| 18 | CnSSR33 | 0.58 | 40 | 5 | 0.60 | 0.35 | 0.55 |
| 19 | CnSSR34 | 0.55 | 40 | 3 | 0.59 | 0.33 | 0.52 |
| 20 | CnSSR35 | 0.75 | 40 | 3 | 0.40 | 0.15 | 0.35 |
| 21 | CnSSR36 | 0.65 | 40 | 4 | 0.52 | 0.30 | 0.46 |
| 22 | CnSSR37 | 0.61 | 40 | 3 | 0.48 | 0.13 | 0.38 |
| 23 | CnSSR38 | 0.66 | 40 | 2 | 0.45 | 0.18 | 0.35 |
| 24 | CnSSR46 | 0.66 | 40 | 3 | 0.50 | 0.20 | 0.45 |
| 25 | CnSSR47 | 0.71 | 40 | 3 | 0.44 | 0.53 | 0.38 |
| 26 | CnSSR48 | 0.56 | 40 | 4 | 0.56 | 0.35 | 0.48 |
| 27 | CnSSR49 | 0.53 | 40 | 2 | 0.50 | 0.30 | 0.37 |
| 28 | CnSSR50 | 0.63 | 40 | 2 | 0.47 | 0.00 | 0.36 |
| 29 | CnSSR51 | 0.39 | 37 | 3 | 0.65 | 0.30 | 0.58 |
| 30 | CnSSR52 | 0.50 | 40 | 2 | 0.50 | 0.15 | 0.38 |
| 31 | CnSSR53 | 0.81 | 40 | 4 | 0.32 | 0.23 | 0.30 |
| 32 | CnSSR54 | 0.59 | 40 | 3 | 0.54 | 0.18 | 0.46 |
| 33 | CnSSR56 | 0.46 | 39 | 4 | 0.64 | 0.03 | 0.57 |
| 34 | CnSSR57 | 0.68 | 40 | 2 | 0.44 | 0.25 | 0.34 |
| 35 | CnSSR58 | 0.74 | 38 | 3 | 0.42 | 0.34 | 0.37 |
| 36 | CnSSR63 | 0.49 | 40 | 3 | 0.58 | 0.23 | 0.49 |
| 37 | CnSSR64 | 0.60 | 35 | 2 | 0.48 | 0.34 | 0.36 |
| 38 | CnSSR67 | 0.77 | 39 | 3 | 0.36 | 0.26 | 0.30 |
| 39 | CnSSR75 | 0.73 | 40 | 5 | 0.44 | 0.30 | 0.40 |
| 40 | CnSSR77 | 0.78 | 40 | 2 | 0.35 | 0.10 | 0.29 |
| 41 | CnSSR78 | 0.87 | 39 | 5 | 0.24 | 0.13 | 0.23 |
| 42 | CnSSR84 | 0.65 | 39 | 2 | 0.45 | 0.13 | 0.35 |
| 43 | CnSSR85 | 0.78 | 39 | 5 | 0.36 | 0.28 | 0.32 |
| 44 | CnSSR87 | 0.79 | 40 | 5 | 0.36 | 0.25 | 0.33 |
| 45 | CnSSR92 | 0.64 | 40 | 3 | 0.53 | 0.23 | 0.47 |
| 46 | CnSSR93 | 0.44 | 40 | 4 | 0.69 | 0.33 | 0.63 |
| 47 | CnSSR97 | 0.69 | 40 | 3 | 0.47 | 0.58 | 0.41 |
| 48 | CnSSR98 | 0.54 | 40 | 4 | 0.59 | 0.30 | 0.52 |
| 49 | CnSSR99 | 0.81 | 39 | 2 | 0.31 | 0.18 | 0.26 |
