# Supplementary material for: Mining and validation of novel genotyping-by-sequencing (GBS)-based simple sequence repeats (SSRs) and their application for the estimation of the genetic diversity and population structure of coconuts (Cocos nucifera L.) in Thailand
Source: Hortic Res. 2020 Oct 1;7:156. doi: 10.1038/s41438-020-00374-1 (PMC7527488; doi:10.1038/s41438-020-00374-1)
Supplement: Supplementary file 6 — Supplementary Figure S1 [file 41438_2020_374_MOESM6_ESM.pdf]

Supplementary Fig. S1 PAGE results for 100 SSR markers. Sizes of uppermost and lowermost alleles in base pair (bp) for each marker are specified.

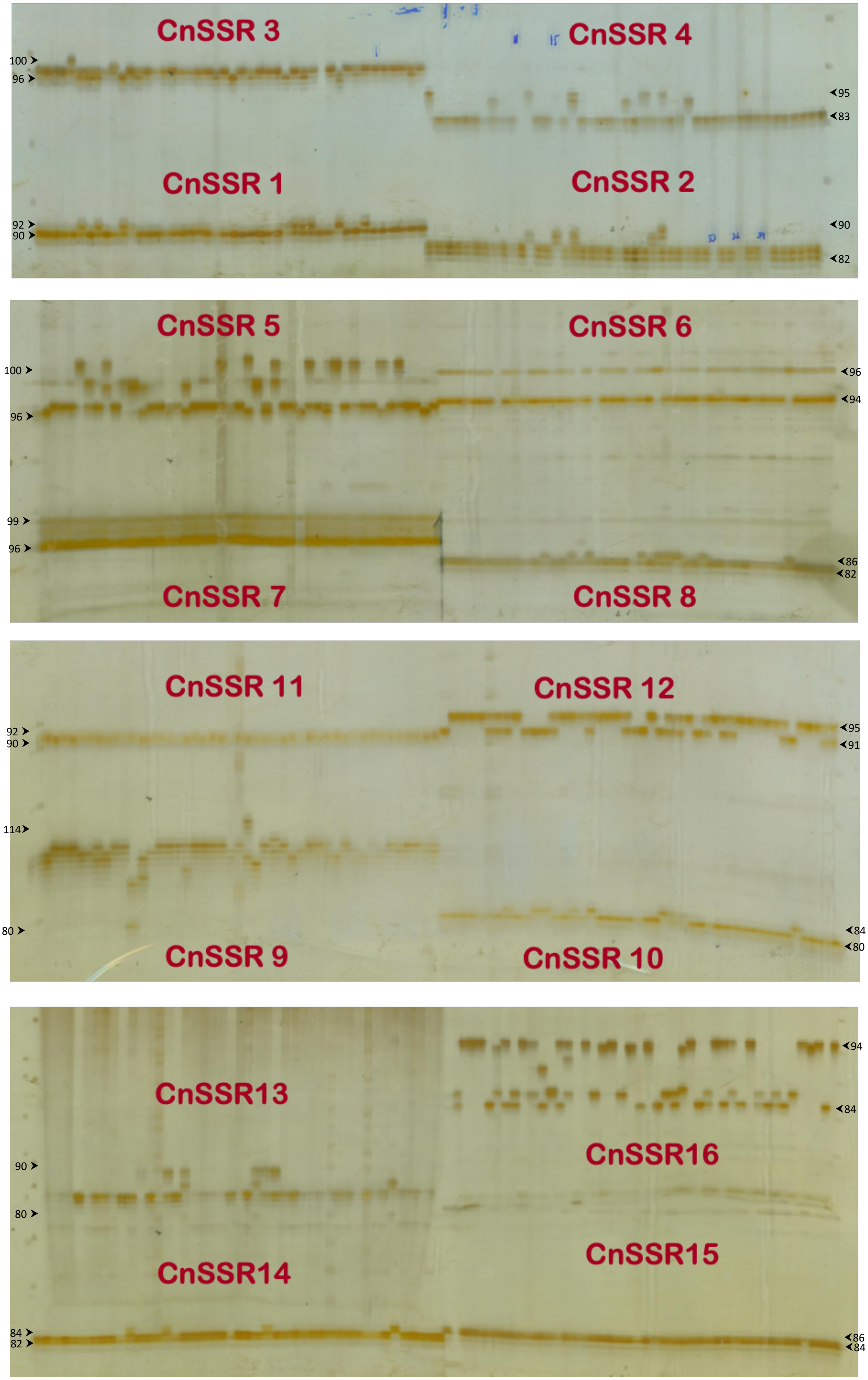

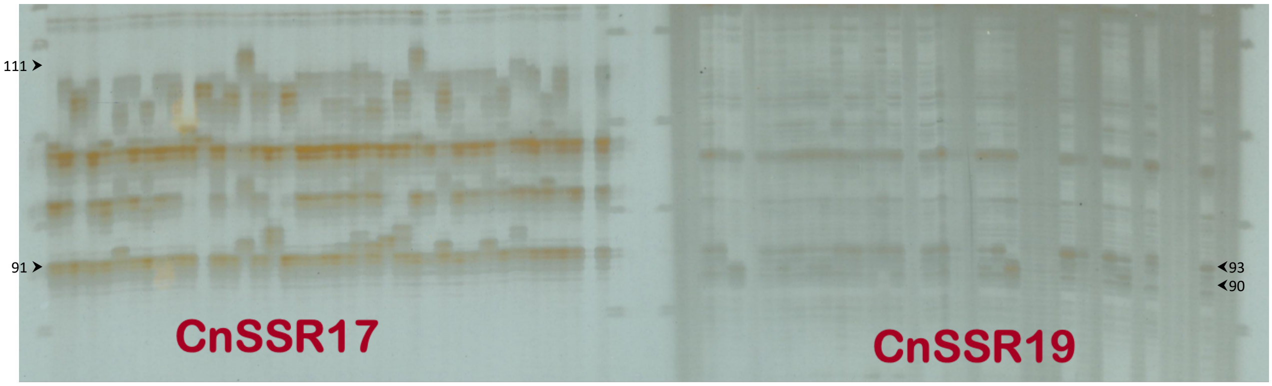
111
91
CnSSR17
CnSSR19
93
90

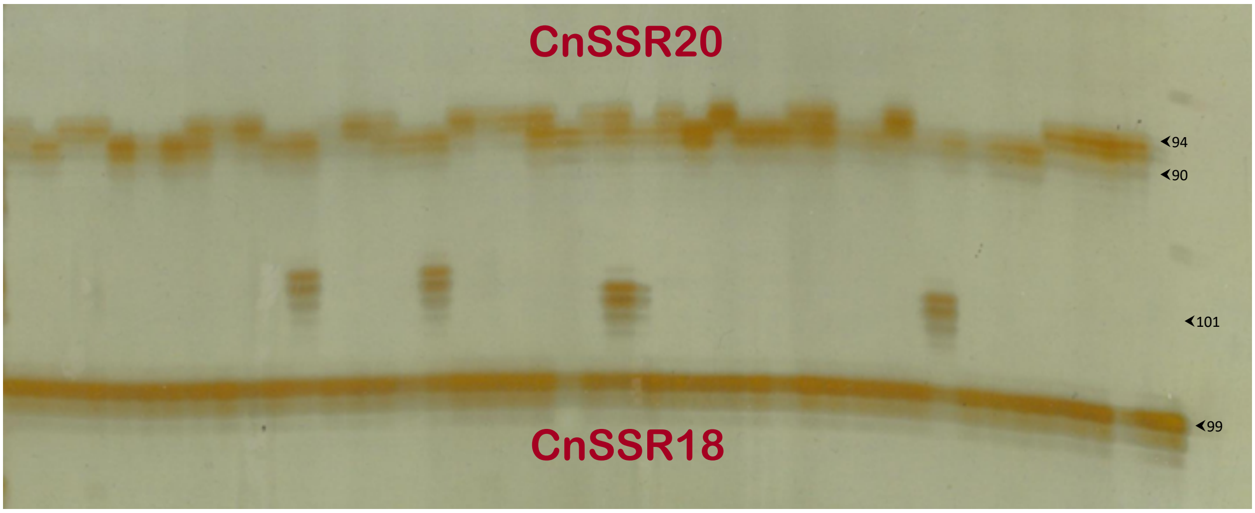
CnSSR20
94
90
101
99
CnSSR18

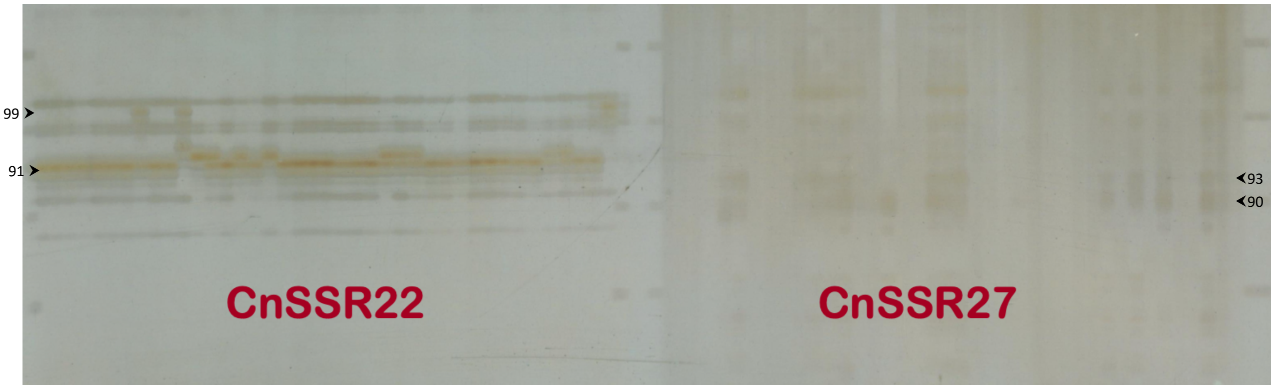
99
91
CnSSR22
CnSSR27
93
90

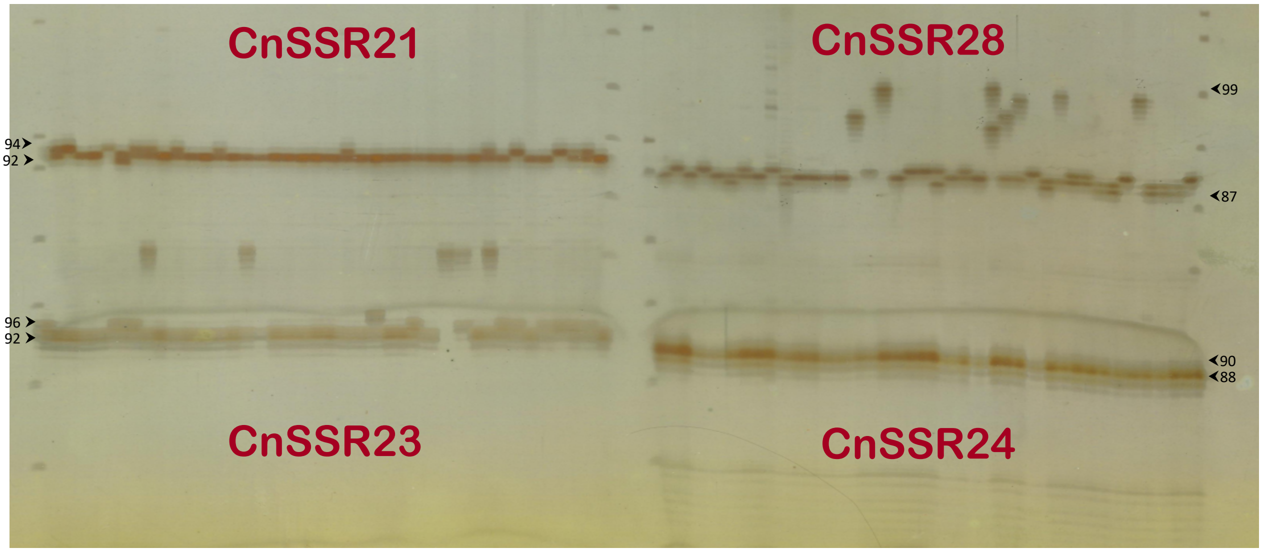
CnSSR21
CnSSR28
99
94
92
87
96
92
90
88
CnSSR23
CnSSR24

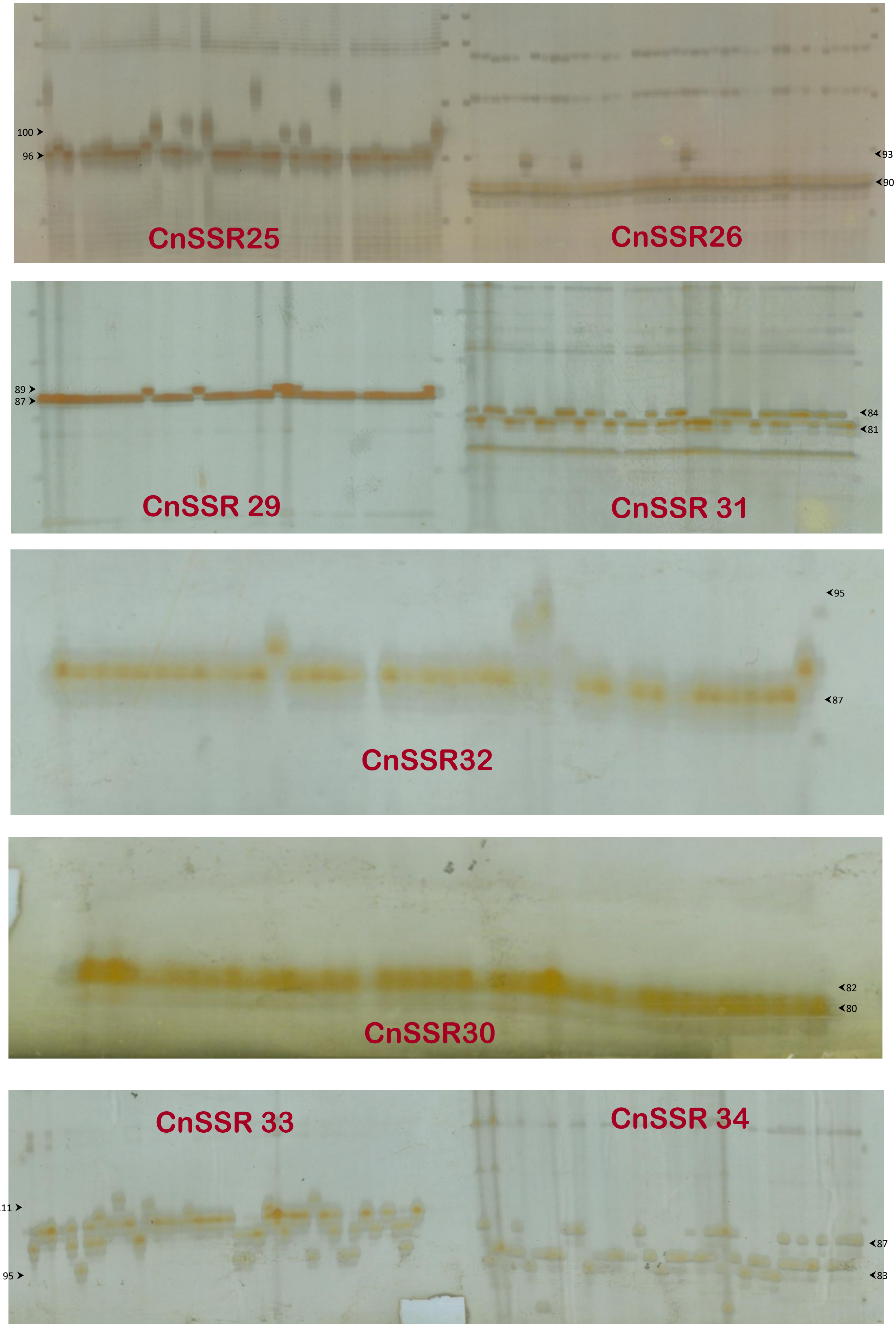
100
96
CnSSR25
93
90
CnSSR26
89
87
CnSSR 29
84
81
CnSSR 31
95
87
CnSSR32
82
80
CnSSR30
CnSSR 33
111
95
CnSSR 34
87
83

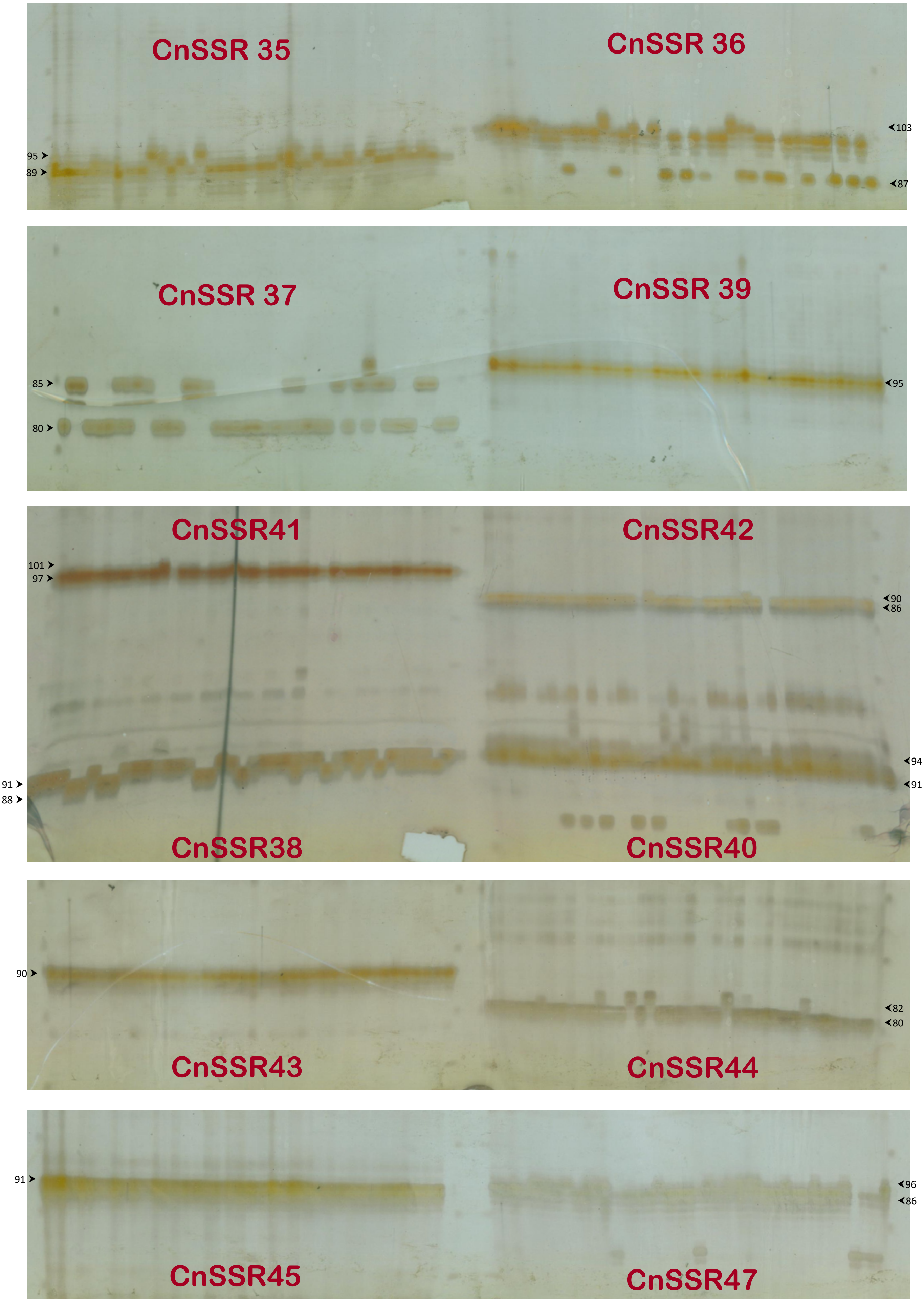
CnSSR 35
CnSSR 36
95
89
103
87
CnSSR 37
CnSSR 39
85
80
95
CnSSR41
CnSSR42
101
97
90
86
94
91
88
91
CnSSR38
CnSSR40
CnSSR43
CnSSR44
90
82
80
CnSSR45
CnSSR47
91
96
86

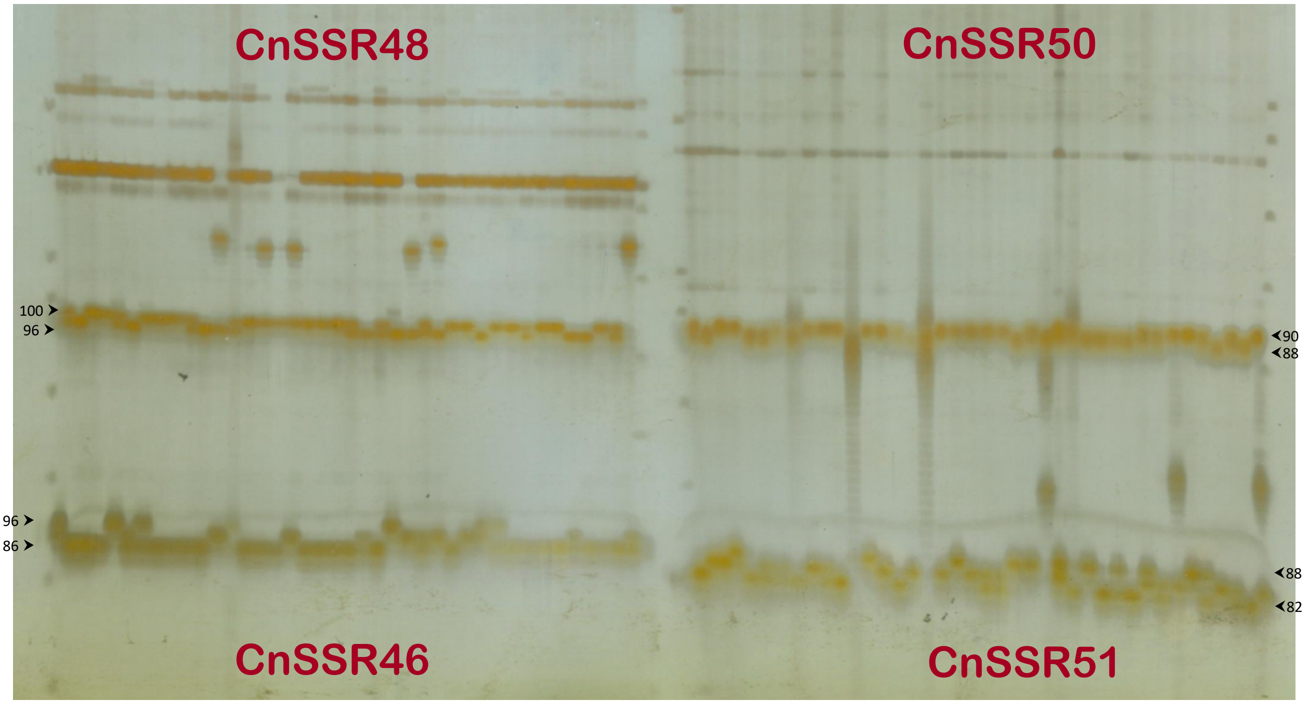
CnSSR48
CnSSR50
100
96
90
88
96
86
88
82
CnSSR46
CnSSR51

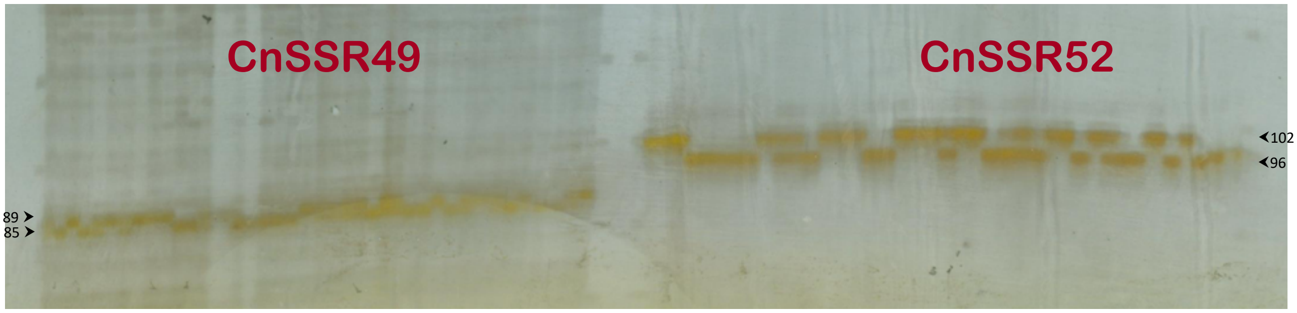
CnSSR49
CnSSR52
102
96
89
85

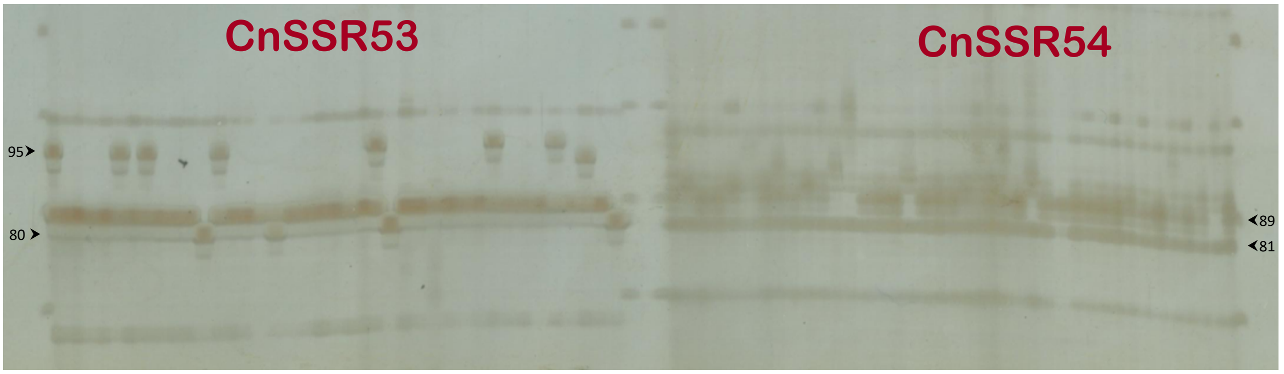
CnSSR53
CnSSR54
95
89
80
81

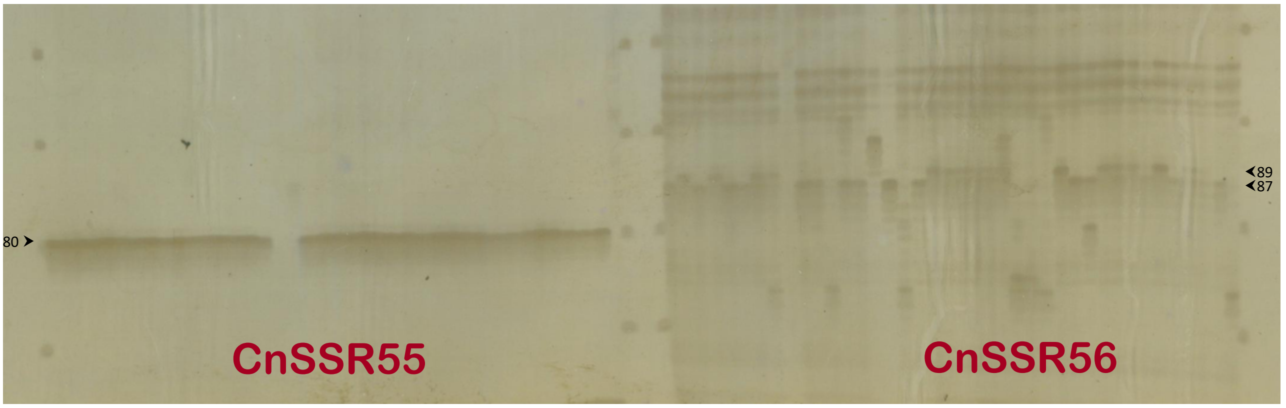
89
87
80
CnSSR55
CnSSR56

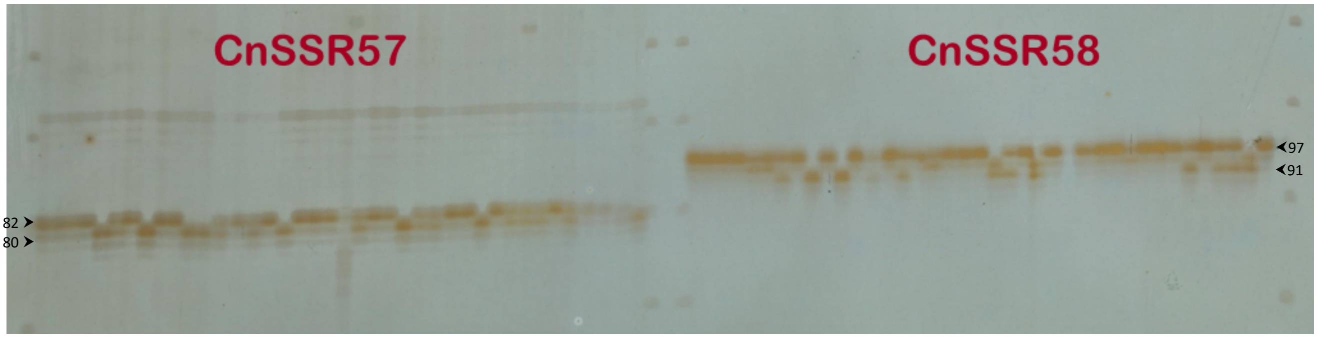
CnSSR57
CnSSR58
82
80
97
91

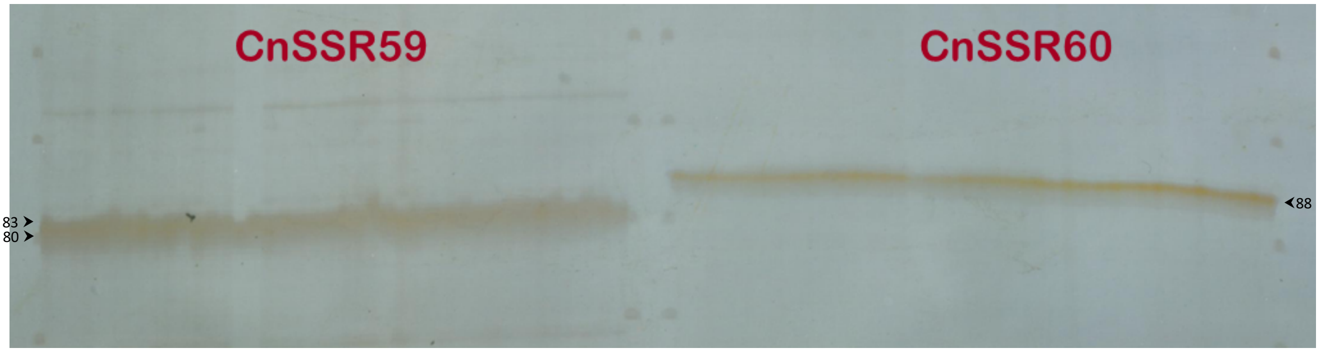
CnSSR59
CnSSR60
83
80
88

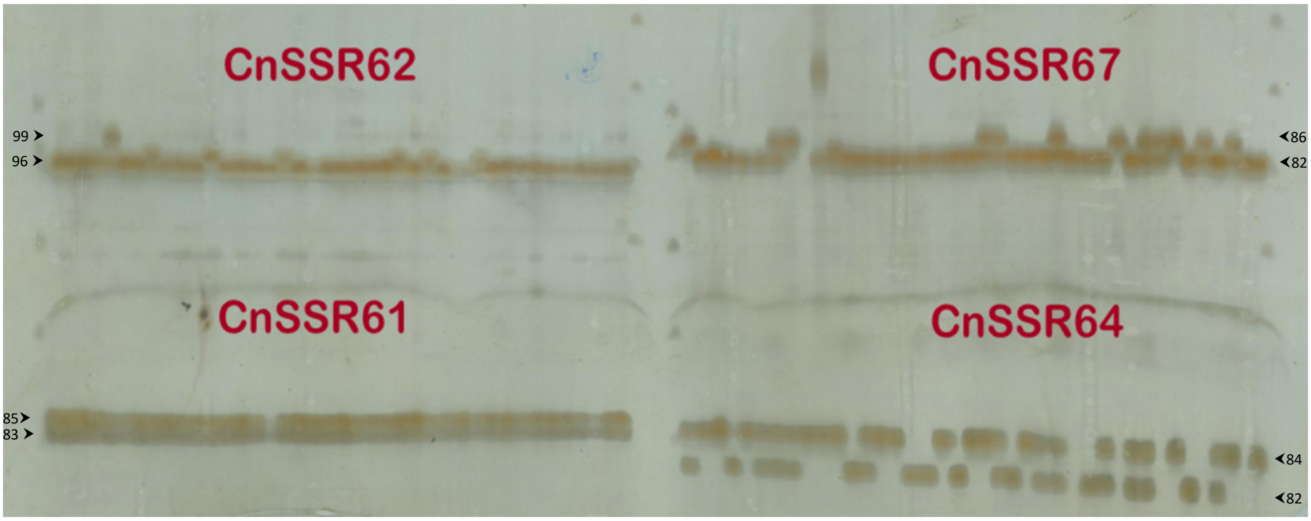
CnSSR62
CnSSR67
99
96
86
82
CnSSR61
CnSSR64
85
83
84
82

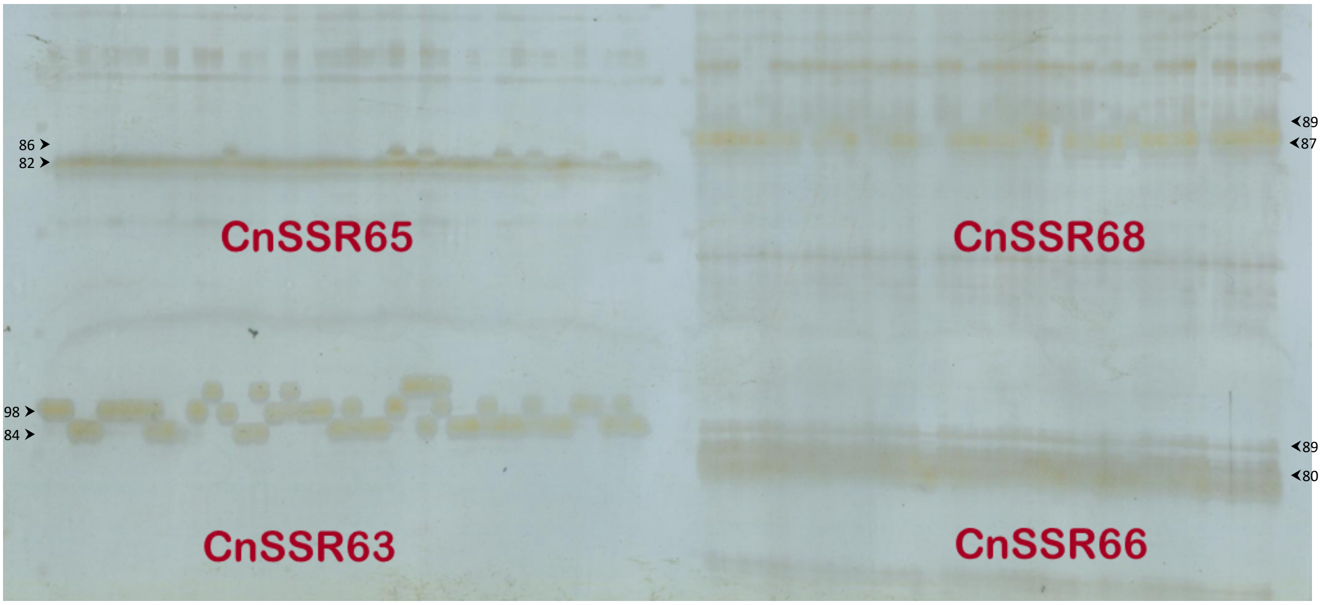
CnSSR65
CnSSR68
86
82
89
87
98
84
CnSSR63
CnSSR66
89
80

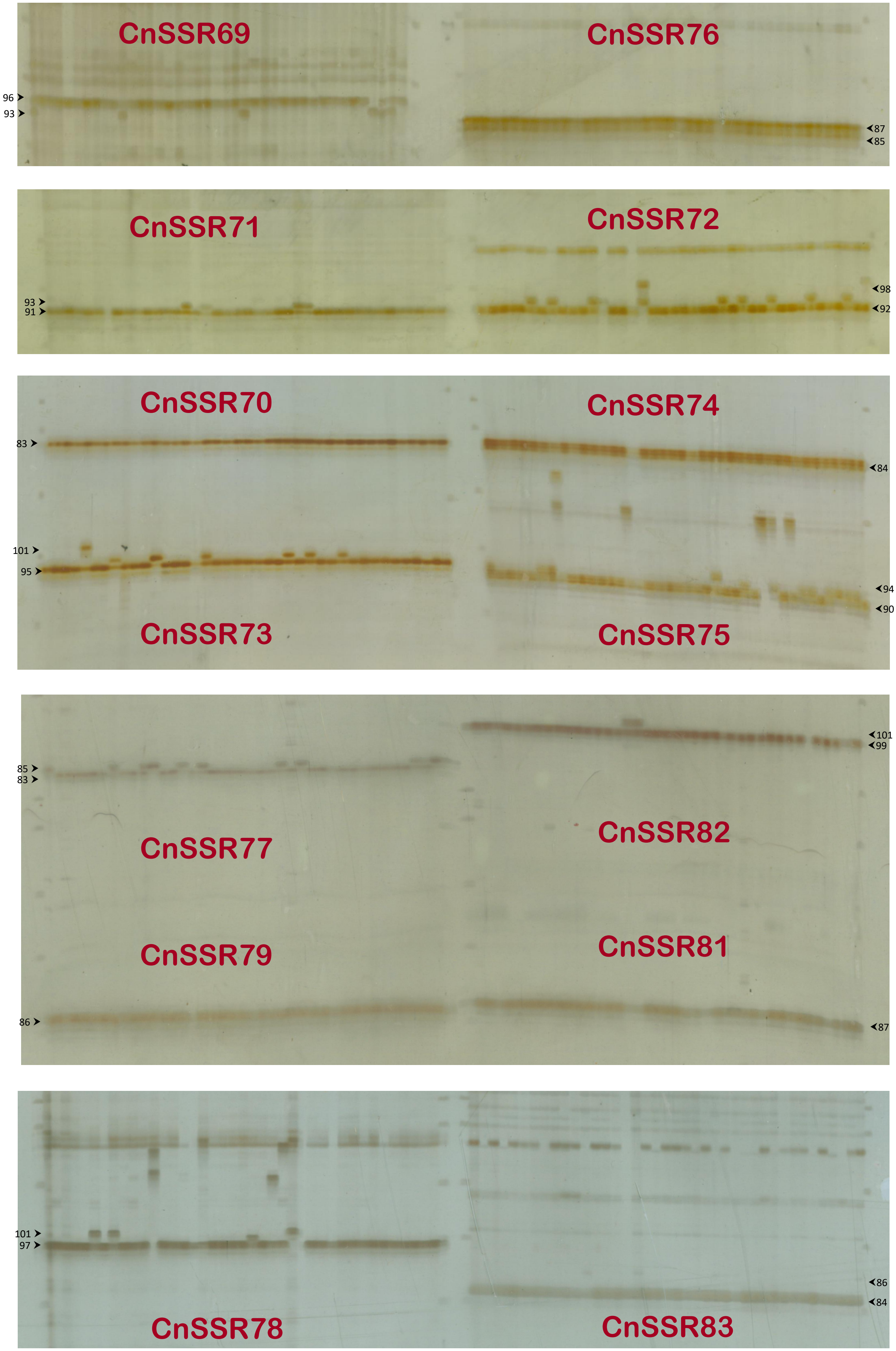
CnSSR69
CnSSR76
96
93
87
85
CnSSR71
CnSSR72
93
91
98
92
CnSSR70
CnSSR74
83
84
101
95
94
90
CnSSR73
CnSSR75
101
99
85
83
CnSSR82
CnSSR77
CnSSR79
CnSSR81
86
87
101
97
86
84
CnSSR78
CnSSR83

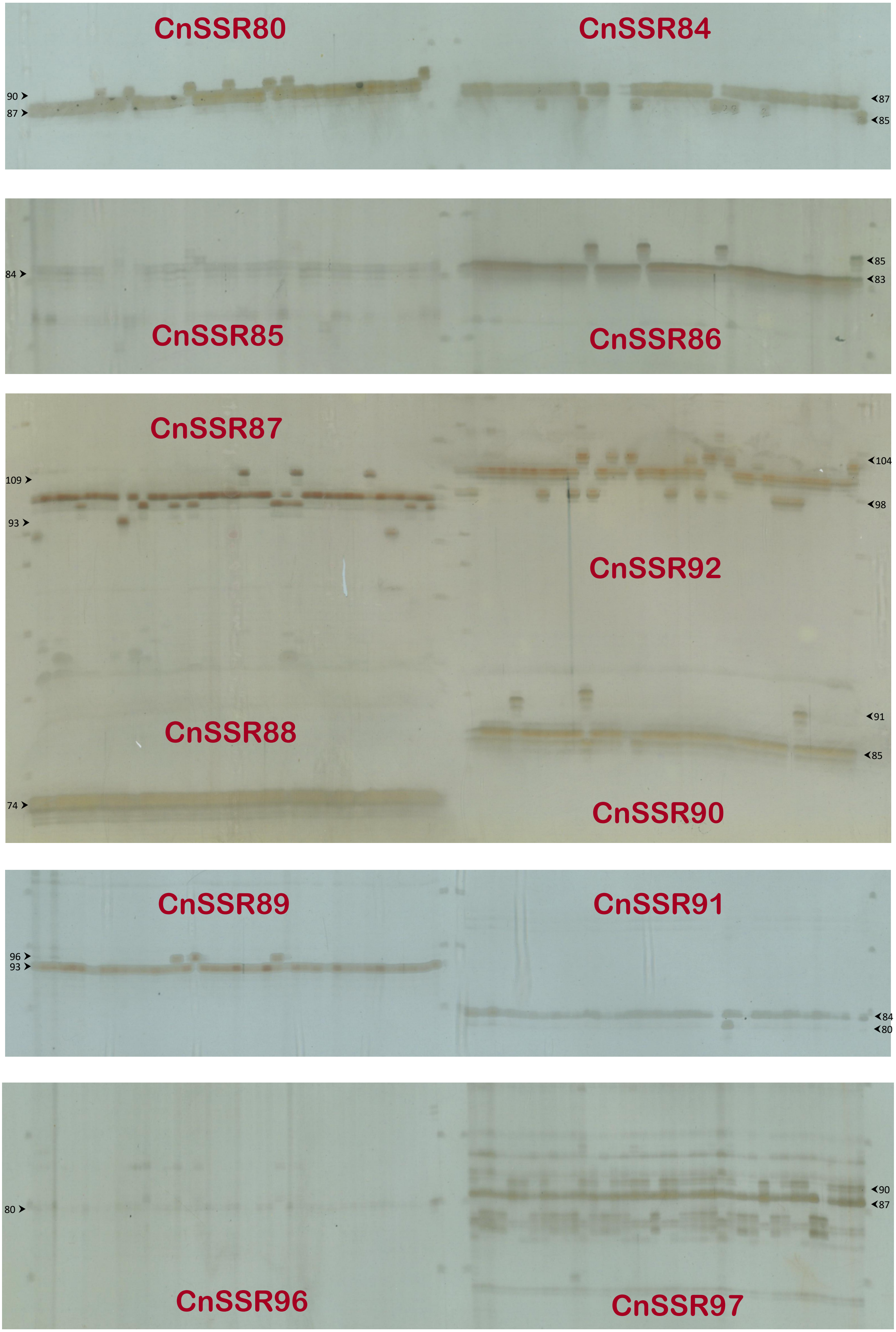
CnSSR80
CnSSR84
90
87
87
85
CnSSR85
CnSSR86
84
85
83
CnSSR87
CnSSR92
109
93
104
98
CnSSR88
CnSSR90
74
91
85
CnSSR89
CnSSR91
96
93
84
80
CnSSR96
CnSSR97
80
90
87

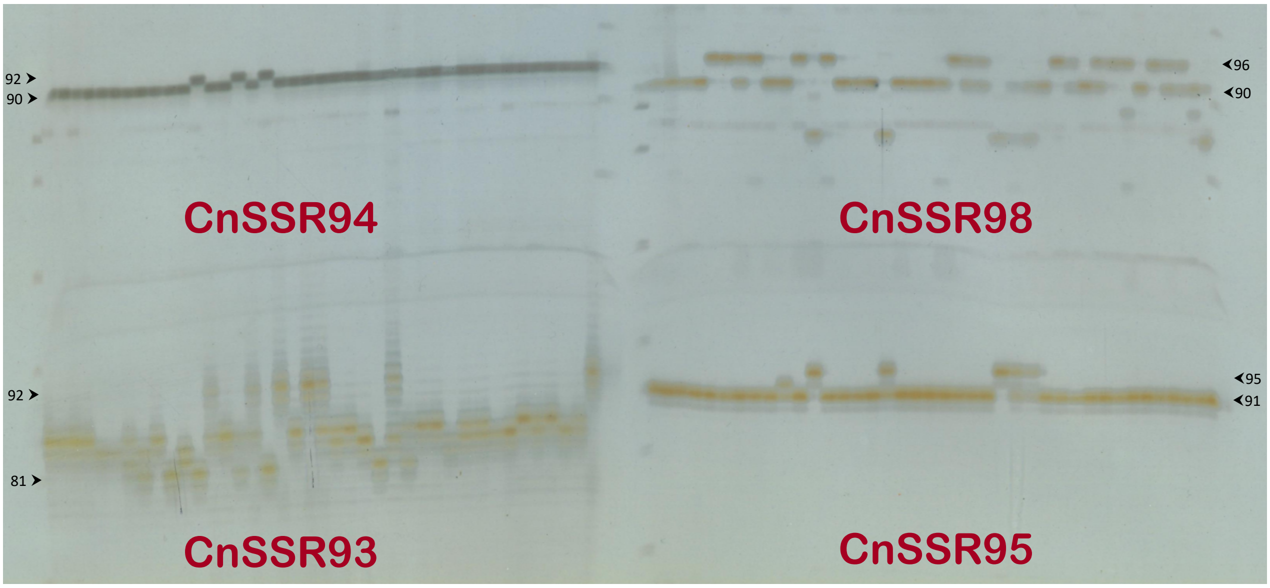
92
90
CnSSR94
CnSSR98
96
90
92
81
CnSSR93
CnSSR95
95
91

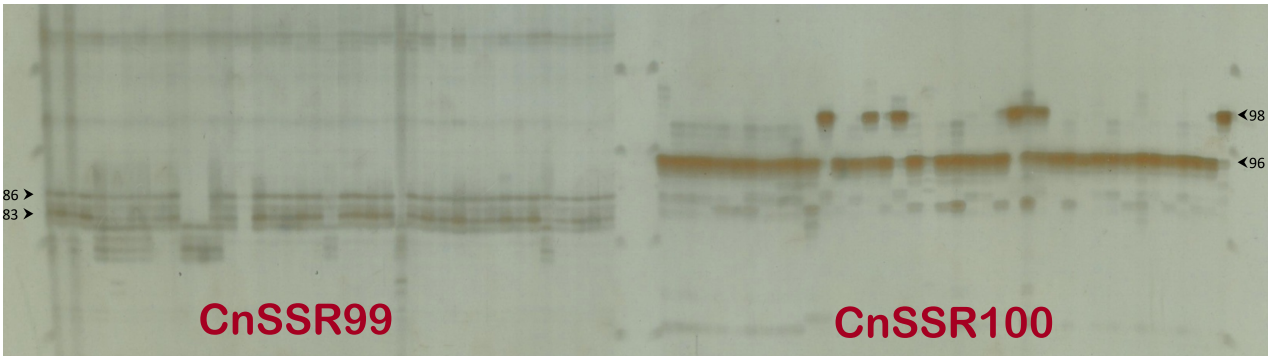
86
83
CnSSR99
CnSSR100
98
96
