# Supplementary material for: Mining and validation of novel genotyping-by-sequencing (GBS)-based simple sequence repeats (SSRs) and their application for the estimation of the genetic diversity and population structure of coconuts (Cocos nucifera L.) in Thailand
Source: Hortic Res. 2020 Oct 1;7:156. doi: 10.1038/s41438-020-00374-1 (PMC7527488; doi:10.1038/s41438-020-00374-1)

Supplementary Fig. S2 Population structure of 40 coconut accessions based on 74 SSR markers. a. Population assignment for each accession estimated at K=2, 3, 4 and 5. b. Plot of the maximum of adhoc statistic ΔK determined by using Structure Harvester. ΔK was calculated for K ranging from 1 to 8.

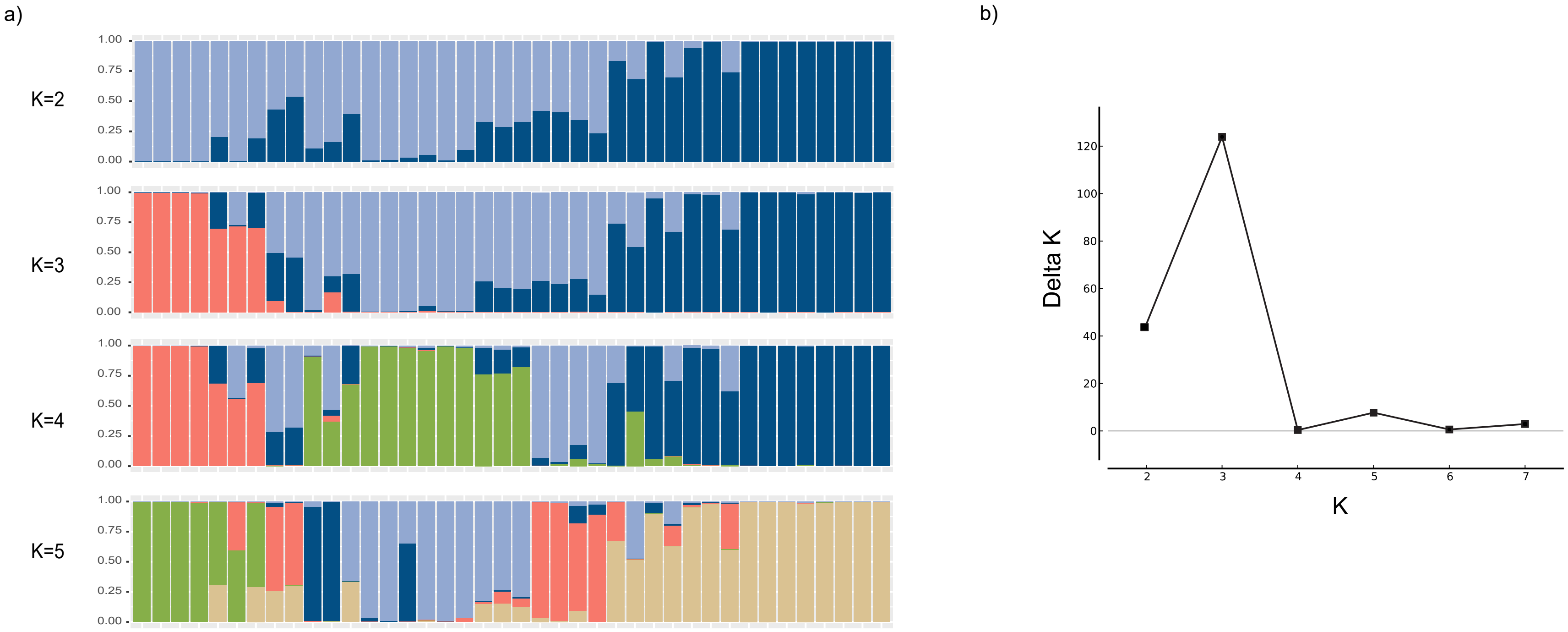

Supplement: Supplementary file 7 — Supplementary Figure S2 [file 41438_2020_374_MOESM7_ESM.pdf]
