# Supplementary material for: Mining and validation of novel genotyping-by-sequencing (GBS)-based simple sequence repeats (SSRs) and their application for the estimation of the genetic diversity and population structure of coconuts (Cocos nucifera L.) in Thailand
Source: Hortic Res. 2020 Oct 1;7:156. doi: 10.1038/s41438-020-00374-1 (PMC7527488; doi:10.1038/s41438-020-00374-1)

Supplementary Fig. S3 PCoA and phylogenetic tree of 40 coconut accessions examined by 49 subset of 74 SSR markers.

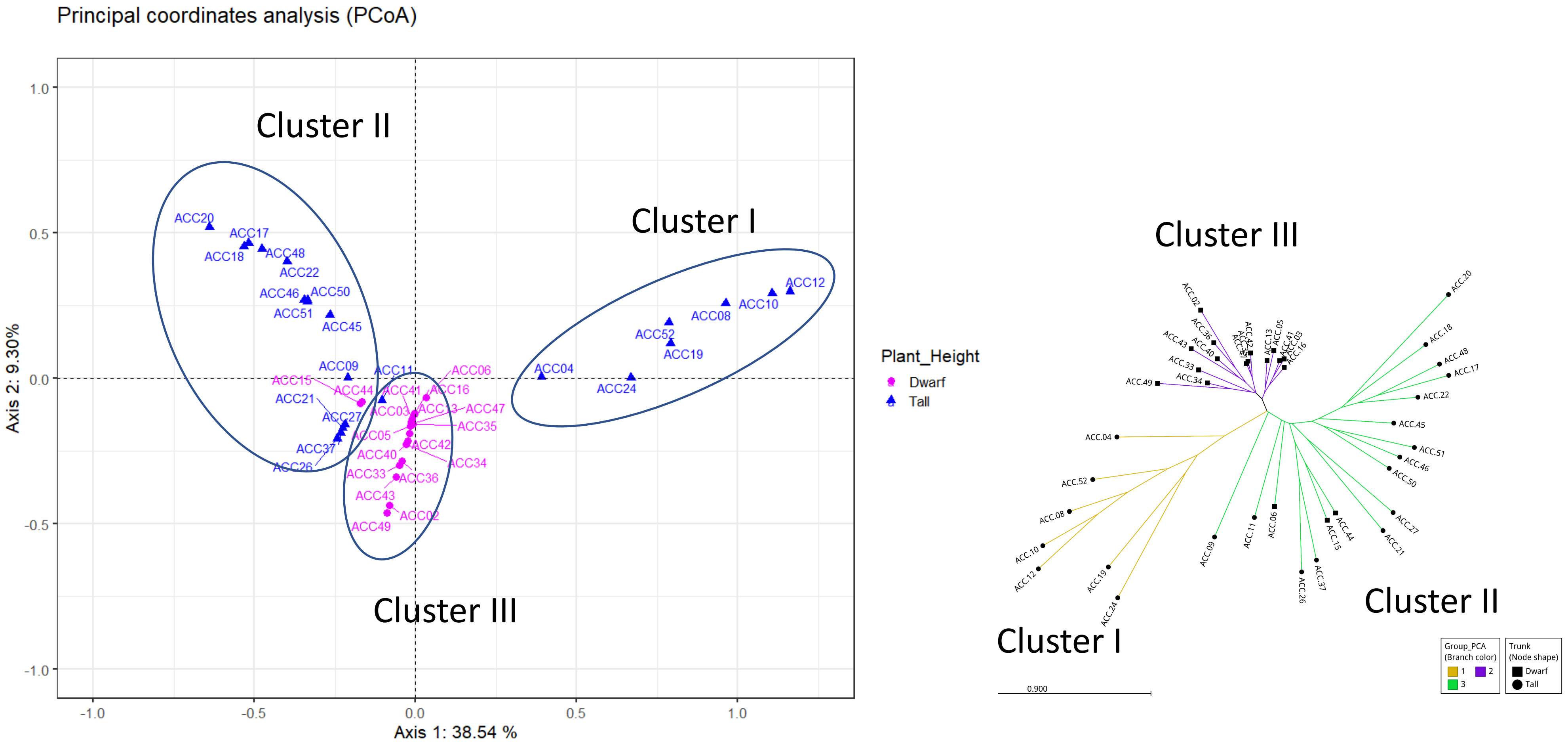

Supplement: Supplementary file 8 — Supplementary Figure S3 [file 41438_2020_374_MOESM8_ESM.pdf]
