# Supplementary material for: Mining and validation of novel genotyping-by-sequencing (GBS)-based simple sequence repeats (SSRs) and their application for the estimation of the genetic diversity and population structure of coconuts (Cocos nucifera L.) in Thailand
Source: Hortic Res. 2020 Oct 1;7:156. doi: 10.1038/s41438-020-00374-1 (PMC7527488; doi:10.1038/s41438-020-00374-1)

Supplementary Fig. S4 Discriminant analysis of principal components (DAPC) for 40 coconut accessions.

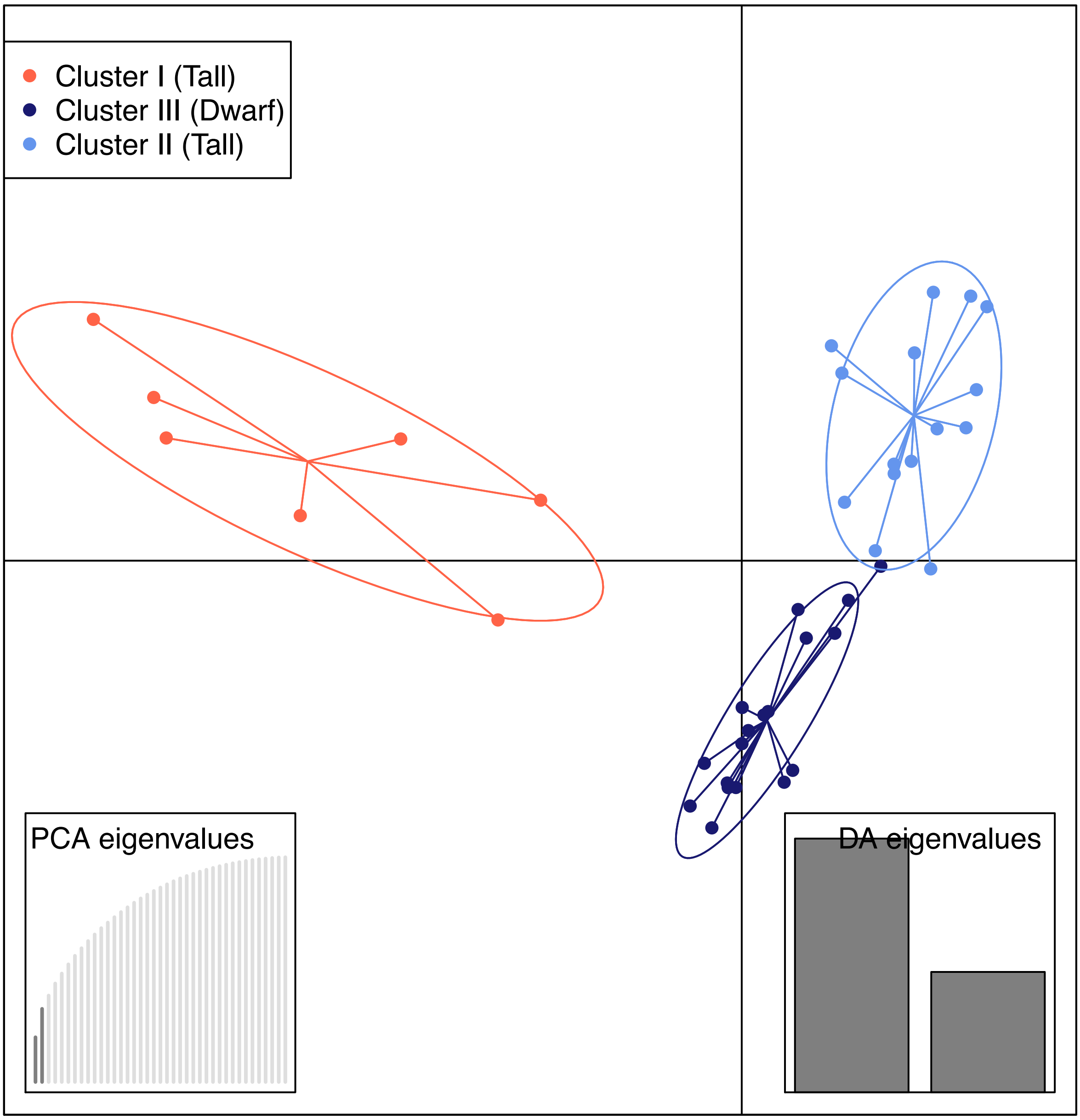

Supplement: Supplementary file 9 — Supplementary Figure S4 [file 41438_2020_374_MOESM9_ESM.pdf]
