# Supplementary material for: Mining and validation of novel genotyping-by-sequencing (GBS)-based simple sequence repeats (SSRs) and their application for the estimation of the genetic diversity and population structure of coconuts (Cocos nucifera L.) in Thailand
Source: Hortic Res. 2020 Oct 1;7:156. doi: 10.1038/s41438-020-00374-1 (PMC7527488; doi:10.1038/s41438-020-00374-1)

Supplementary Fig. S5 (a) Locations on the map where coconut accessions were sampled. (b) The correlation between pairwise genetic distance and geographic distance among each Thai coconut accession.

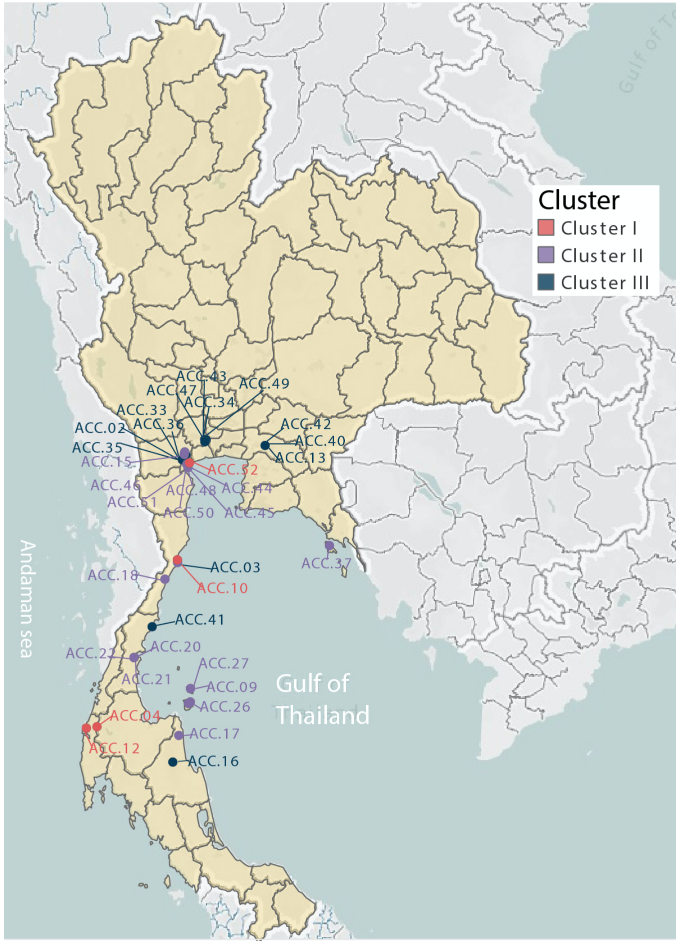

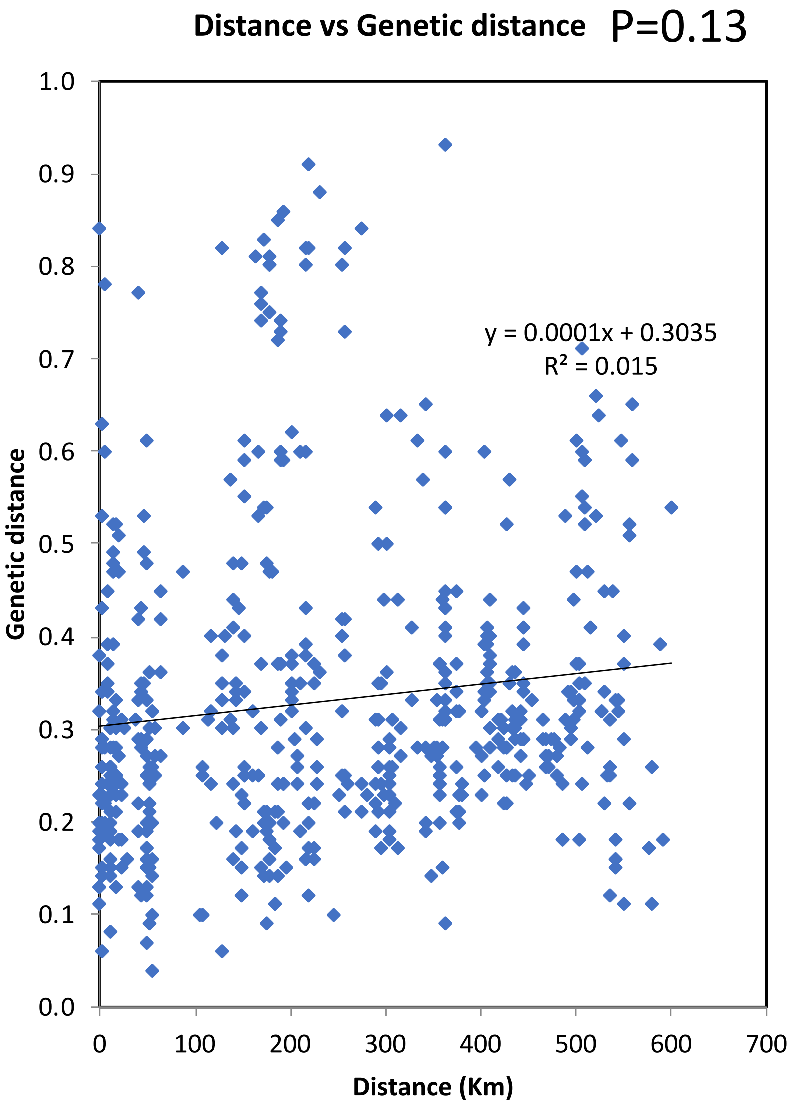

Supplement: Supplementary file 10 — Supplementary Figure S5 [file 41438_2020_374_MOESM10_ESM.pdf]
